# Quantifying the Excess Risk of Adverse COVID-19 Outcomes in Unvaccinated Individuals With Diabetes Mellitus, Hypertension, Ischaemic Heart Disease or Myocardial Injury: A Meta-Analysis

**DOI:** 10.3389/fcvm.2022.871151

**Published:** 2022-04-26

**Authors:** Sher May Ng, Jiliu Pan, Kyriacos Mouyis, Sreenivasa Rao Kondapally Seshasai, Vikas Kapil, Kenneth M. Rice, Ajay K. Gupta

**Affiliations:** ^1^St. Bartholomew's Hospital, London, United Kingdom; ^2^Royal Brompton and Harefield Hospitals, London, United Kingdom; ^3^Royal Free London NHS Foundation Trust, London, United Kingdom; ^4^Cardiovascular Clinical Academic Group, Molecular and Clinical Sciences Research Institute, St. George's University of London, St. George's University Hospitals NHS Foundation Trust, London, United Kingdom; ^5^William Harvey Research Institute, Queen Mary University London, London, United Kingdom; ^6^Department of Biostatistics, University of Washington, Seattle, WA, United States

**Keywords:** COVID-19, cardiovascular risk factors, myocardial injury, ischaemic heart disease, diabetes, hypertension, adverse outcomes

## Abstract

**Background:**

More than 80% of individuals in low and middle-income countries (LMICs) are unvaccinated against coronavirus disease 2019 (COVID-19). In contrast, the greatest burden of cardiovascular disease is seen in LMIC populations. Hypertension (HTN), diabetes mellitus (DM), ischaemic heart disease (IHD) and myocardial injury have been variably associated with adverse COVID-19 outcomes. A systematic comparison of their impact on specific COVID-19 outcomes is lacking. We quantified the impact of DM, HTN, IHD and myocardial injury on six adverse COVID-19 outcomes: death, acute respiratory distress syndrome (ARDS), invasive mechanical ventilation (IMV), admission to intensive care (ITUadm), acute kidney injury (AKI) and severe COVID-19 disease (SCov), in an unvaccinated population.

**Methodology:**

We included studies published between 1^st^ December 2019 and 16^th^ July 2020 with extractable data on patients ≥18 years of age with suspected or confirmed SARS-CoV-2 infection. Odds ratios (OR) for the association between DM, HTN, IHD and myocardial injury with each of six COVID-19 outcomes were measured.

**Results:**

We included 110 studies comprising 48,809 COVID-19 patients. Myocardial injury had the strongest association for all six adverse COVID-19 outcomes [death: OR 8.85 95% CI (8.08–9.68), ARDS: 5.70 (4.48–7.24), IMV: 3.42 (2.92–4.01), ITUadm: 4.85 (3.94–6.05), AKI: 10.49 (6.55–16.78), SCov: 5.10 (4.26–6.05)]. HTN and DM were also significantly associated with death, ARDS, ITUadm, AKI and SCov. There was substantial heterogeneity in the results, partly explained by differences in age, gender, geographical region and recruitment period.

**Conclusion:**

COVID-19 patients with myocardial injury are at substantially greater risk of death, severe disease and other adverse outcomes. Weaker, yet significant associations are present in patients with HTN, DM and IHD. Quantifying these associations is important for risk stratification, resource allocation and urgency in vaccinating these populations.

**Systematic Review Registration:**

https://www.crd.york.ac.uk/prospero/, registration no: CRD42020201435 and CRD42020201443.

## Introduction

The global coronavirus disease 2019 (COVID-19) pandemic has impacted healthcare systems and economies worldwide. It has laid bare health inequalities and magnified unequal effects of public health measures implemented across the world, with associated ramifications on global health. This is well-exemplified by the global COVID-19 vaccine inequality, where only 14.4% of individuals in low-income countries have received one dose of COVID-19 vaccine, as of March 2022 (1).

Low and middle-income countries (LMICs) are plagued by the difficult decision between strict non-pharmaceutical interventions such as national lockdowns and their socioeconomic impact, particularly on the urban poor. Moreover, these countries suffer from increased COVID-19 associated mortality owing to insufficient healthcare resources and poorly funded emergency response programmes (2, 3).

While high-income countries (HICs) have made significant progress in vaccination rollout programmes, LMICs continue to lag behind. Importantly, studies from HIC populations have suggested the COVID-19 incidence and hospitalization rates among unvaccinated individuals are approximately 2 and 5-times that of vaccinated (but without a booster) individuals respectively (4).

Multiple observational studies have shown associations between cardiovascular (CV) risk factors such as hypertension (HTN), diabetes mellitus (DM), previous ischaemic heart disease (IHD), myocardial injury and outcomes such as mortality or severe disease due to COVID-19 (SCov). Other studies have challenged these findings, showing heterogeneous associations between these risk factors and COVID-19 related death (5, 6). In addition, there remains a lack of consensus on the impact of myocardial injury and CV risk factors on other important COVID-19 adverse outcomes such as acute respiratory distress syndrome (ARDS), invasive mechanical ventilation (IMV) and intensive care admission (ITUadm).

Therefore, quantifying the risk of COVID-19-related adverse outcomes attributable to CV risk factors, IHD and myocardial injury in unvaccinated persons is essential, not only for patient-specific care but also for risk stratification and planning of healthcare delivery in already stretched LMICs. This is especially relevant where the greatest burden of cardiovascular disease is amongst LMICs. In this meta-analysis, we quantify the association between CV risk factors, IHD and myocardial injury, and specific adverse clinical outcomes in unvaccinated adults with COVID-19 infection.

## Methods

The protocol for this meta-analysis with pre-specified aims and objectives was prospectively registered on PROSPERO (CRD42020201435 and CRD42020201443). The aim of the meta-analysis was to study the impact of pre-specified cardiovascular risk factors (HTN, DM, previous IHD, and presence of myocardial injury) on adverse COVID-19 outcomes [all-cause mortality, ARDS, IMV, admission to intensive care (ITUadm), AKI, and study-defined severe COVID-19 disease (SCoV)]. We included studies published (in print or pre-print version) during the early phase of the COVID-19 pandemic, prior to widespread use of COVID-19 vaccinations.

### Population Selection

We included studies that reported prevalence of pre-existing cardiovascular risk factors in adult patients (≥18 years of age) with suspected or confirmed COVID-19 disease and any one of the COVID-19 related adverse outcomes.

### Search Strategy

We searched databases of published (MEDLINE, CINAHL, Embase, EMCARE, British Nursing Index) and pre-print (medRxiv) articles without language restrictions, between 1st December 2019 and 16th July 2020. We also searched data from the COVID-19 specific World Health Organization (WHO) global research database. Duplicate studies were identified and removed initially through a Mendeley folder (AT) followed by manual de-duplication by two authors working independently (JP and SMN). The final study list was agreed by consensus. Three authors (JP, KM, SMN) screened references of full-text studies, review articles and existing meta-analyses for additional studies. Appendix A gives the full search strategy.

### Selection Criteria

**Figure 1:**
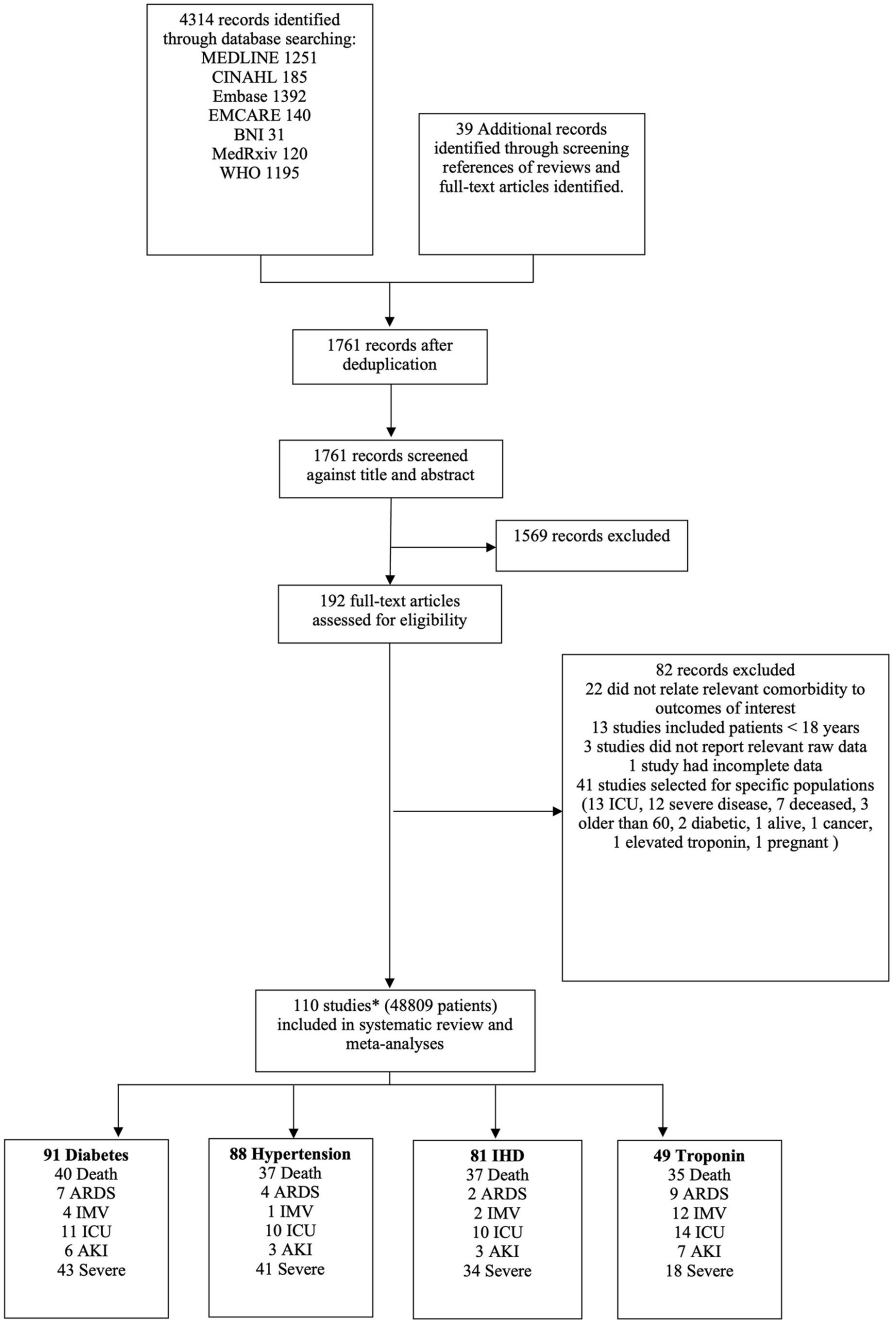
PRISMA flow chart of study selection. *Three studies reported different outcomes on the same cohort. Therefore, 208 patients have been excluded from the total number of patients as they were considered to be a duplicate cohort.

Studies were included if they had extractable data on (i) patients ≥ 18 years of age with suspected or confirmed SARS-CoV-2 infection (COVID-19); (ii) pre-existing CV risk factors, specifically HTN and DM, IHD, or evidence of myocardial injury (defined as serum troponin level above the 99th percentile upper reference limit); and (iii) COVID-19 related outcomes, in particular all-cause mortality, ARDS, IMV, admission to intensive care (ITUadm), AKI, and study-defined severe COVID-19 disease (SCoV). Data from Chinese studies were translated and extracted by JP. Studies reporting only on special populations (e.g., dialysis patients, pregnant women, elderly patients or children) were excluded (Appendix C). Case reports, case series, review articles and meta-analyses were also excluded. Figure 1 shows the PRISMA flow chart for study selection.

Three authors (KM, JP, SMN) independently screened all titles and abstracts, reviewed full text articles, extracted data onto pre-specified forms and performed risk of bias assessments. Disagreements were resolved by consensus. Risk of bias assessment for individual studies was performed using the Newcastle-Ottawa Scale (NOS) (Appendix D). The quality of body of evidence for each outcome was assessed using Grading of Recommendations, Assessment, Development and Evaluation (GRADE) working group approach (Appendix E).

### Statistical Analysis

Associations between disease status and COVID-19 outcomes were quantified using odds ratios (OR) which were combined using fixed-effects meta-analysis (7). Random-effects analysis was also performed as an alternative assessment of the impact of heterogeneity on the analyses. Results presented were derived from the fixed-effects model if no notable difference from the random-effects analysis was identified. Heterogeneity around the fixed-effects (inverse-variance weighted) average effect was assessed using the *I*^2^ statistic (8) and where larger numbers of studies were present, fixed-effects meta-regression. Meta-regression was used to assess the effect-modification of disease status on COVID-19 outcomes by age, geographic region, date of last recruitment and proportion of male participants. In sensitivity analyses to address miscalibration of inference due to small sample sizes, we compared meta-analysis results to those with higher-order accuracy (9).

## Results

We identified 110 studies comprising 49,017 patients with COVID-19. The full list of included and excluded studies are detailed in Appendices B, C. As three studies reported on the same cohort of patients, we excluded 208 individuals from the meta-analysis, leaving 48,809 patients (Figure 1). The mean age was 56.7 years and 57% were male. A median of three risk factors and outcomes were reported per study. In total 20% had DM, 37% had HTN, 10% had IHD and 12% had evidence of myocardial injury. Death and severe COVID-19 disease were the commonest reported outcomes; overall, there were 7,150 deaths, 2,180 cases of ARDS, 3,162 individuals needing IMV, 2,950 admissions to intensive care, 3119 patients with AKI and 4,804 severe COVID-19 cases. Table 1 summarizes the characteristics of included studies, categorized by risk factors.

**Table 1:** Characteristics of included studies reporting COVID-19 related outcomes, categorized by risk factors.

**Outcome by risk factor**	**Number of studies**	**Total number of patients**	N patients with risk factor^*^ No. (%)	N patients with outcome^**^ **No. (%)**	**Mean age**	**Male no. (%)**	**Region of study**	**N patients with risk factor and outcome** **No. (%)**	**% of patients exposed to risk factor reaching outcome**
**Death**									
Diabetes mellitus	40	18,979	3,791 (20)	3,194 (17)	60	61	24 Asia, 11 Europe, 5 USA	984 (5)	26
Hypertension	37	17,995	6,695 (37)	3,063 (17)	59.1	60	22 Asia, 10 Europe, 5 USA	1,698 (9)	25
Ischaemic heart disease	37	19,968	2,619 (13)	3,521 (18)	60.3	60	21 Asia, 11 Europe, 5 USA	928 (5)	35
Myocardial injury	35	21,707	5,225 (24)	3,259 (15)	58.2	54	26 Asia, 5 Europe, 4 USA	2,197 (10)	42
**ARDS**									
Diabetes mellitus	7	1,428	257 (18)	404 (28)	57	750 (53)	6 Asia, 1 Europe	112 (8)	44
Hypertension	4	476	154 (32)	172 (36)	56.5	287 (60)	3 Asia, 1 Europe	65 (14)	42
Ischaemic heart disease	2	310	15 (5)	137 (44)	53	187 (60)	2 Asia	8 (3)	53
Myocardial injury	9	2,189	584 (27)	615 (28)	57.8	1,128 (52)	6 Asia, 2 Europe, 1 USA	348 (16)	60
**IMV**									
Diabetes mellitus	4	1,345	275 (20)	214 (16)	58.7	1,376 (52)	3 Asia, 1 USA	65 (5)	24
Hypertension	1	393	197 (50)	130 (33)	61.5	238 (61)	1 USA	70 (18)	36
Ischaemic heart disease	2	8,831	777 (9)	689 (8)	59.6	4,782 (54)	2 USA	109 (1)	14
Myocardial injury	12	10,424	2,796 (26)	836 (8)	57.3	5,625 (54)	9 Asia, 1 Europe, 2 USA	553 (5)	20
**ITU**									
Diabetes mellitus	11	2,487	482 (19)	432 (17)	56.9	1,376 (55)	7 Asia, 3 Europe, 1 USA	133 (5)	28
Hypertension	10	1,891	761 (40)	394 (21)	57.2	1,089 (58)	6 Asia, 3 Europe, 1 USA	211 (11)	28
Ischaemic heart disease	10	1,891	259 (14)	394 (21)	57.2	1,089 (58)	6 Asia, 3 Europe, 1 USA	63 (3)	24
Myocardial injury	14	2,753	698 (25)	644 (23)	56	1,487 (54)	9 Asia, 4 Europe, 1 USA	309 (11)	44
**AKI**									
Diabetes mellitus	6	7,018	2,124 (30)	2,205 (31)	59.6	4,081 (58)	4 Asia, 2 USA	914 (13)	43
Hypertension	3	6,066	3,332 (55)	2,140 (35)	61.5	3,618 (60)	1 Asia, 2 USA	1,419 (23)	43
Ischaemic heart disease	3	6,066	673 (11)	2,140 (35)	61.5	3,618 (60)	1 Asia, 2 USA	318 (5)	47
Myocardial injury	7	1,777	410 (23)	153 (9)	57.4	914 (51)	5 Asia, 1 Europe, 1 USA	113 (6)	28
**Severe disease**									
Diabetes mellitus	43	11,495	2,171 (19)	3,444 (30)	52.3	6,215 (54)	41 Asia, 2 USA	959 (8)	44
Hypertension	41	10,653	3,774 (35)	3,206 (30)	50.8	5,800 (54)	39 Asia, 2 USA	1,602 (15)	42
Ischaemic heart disease	34	10,149	1,325 (13)	3,001 (30)	53.3	5,531 (54)	32 Asia, 2 USA	644 (6)	48
Myocardial injury	18	4,731	925 (20)	1,461 (31)	53.2	2,456 (52)	16 USA, 1 Europe, 1 USA	549 (12)	59

* *Risk factors are defined as presence of diabetes, hypertension, ischaemic heart disease or myocardial injury. ARDS, acute respiratory distress syndrome; IMV, invasive mechanical ventilation; ITU, admission to intensive care; AKI, acute kidney injury*.

** *Total number of patients with specific outcomes presented in this table include only studies which reported outcomes according to risk factors of interest. Reported outcomes that were not explicitly associated with risk factors are not included in this table. Therefore, these numbers may not reflect the total number of adverse COVID-19 outcomes in all included studies*.

### Presence of Diabetes, Hypertension and Ischaemic Heart Disease and COVID-19 Outcomes

Table 2 summarizes the associations between the four studied risk factors and six outcomes of interest.

**Table 2:** Odds Ratio [Confidence Intervals] for COVID-19 adverse outcomes according to risk exposure.

	**COVID-19 adverse outcome Odds ratio [95% confidence interval]**																	
**Risk exposure**	**Death**	***p-*value**	***I^**2**^***	**ARDS**	***p-*value**	***I^**2**^***	**IMV**	***p*-value**	***I^**2**^***	**ITU**	***p*-value**	***I^**2**^***	**AKI**	***p*-value**	**I^**2**^**	**Severe** **Disease**	**p-value**	**I^**2**^**
Diabetes mellitus	2.15 [1.97, 2.36]	<0.001	68.6	2.48 [1.82, 3.32]	<0.001	43.9	1.77 [1.25, 2.51]	0.0014	66.3	1.65 [ 1.26, 2.16]	<0.001	60.1	1.84 [1.65, 2.05]	<0.001	0.0	1.80 [1.63, 1.99]	<0.001	48.0
Hypertension	2.72 [2.51, 2.97]	<0.001	67.8	1.68 [1.07, 2.63]	0.025	0.0	1.24 [0.82, 1.90]	0.30	N/A	1.55 [1.20, 1.99]	<0.001	66.3	1.90 [1.70, 2.12]	<0.001	88.5	2.14 [1.93, 2.34]	<0.001	73.4
Ischaemic heart disease	3.29 [3.00, 3.63]	<0.001	51.2	1.42 [0.49, 4.10]	0.49	7.1	1.99 [1.58, 2.48]	<0.001	0.0	1.51 [1.05, 2.16]	0.024	69.3	1.75 [1.49, 2.05]	<0.001	37.6	2.20 [1.93, 2.51]	<0.001	40.2
Myocardial injury	8.85 [8.08, 9.68]	<0.001	77.1	5.70 [4.48, 7.24]	<0.001	82.7	3.42 [2.92, 4.01]	<0.001	70.1	4.85 [3.94, 6.05]	<0.001	75.7	10.49 [6.55, 16.78]	<0.001	10.8	5.10 [4.26, 6.05]	<0.001	73.5

*ARDS, acute respiratory distress syndrome; IMV, invasive mechanical ventilation; ITU, admission to intensive care; AKI, acute kidney injury*.

**Figure 2:**
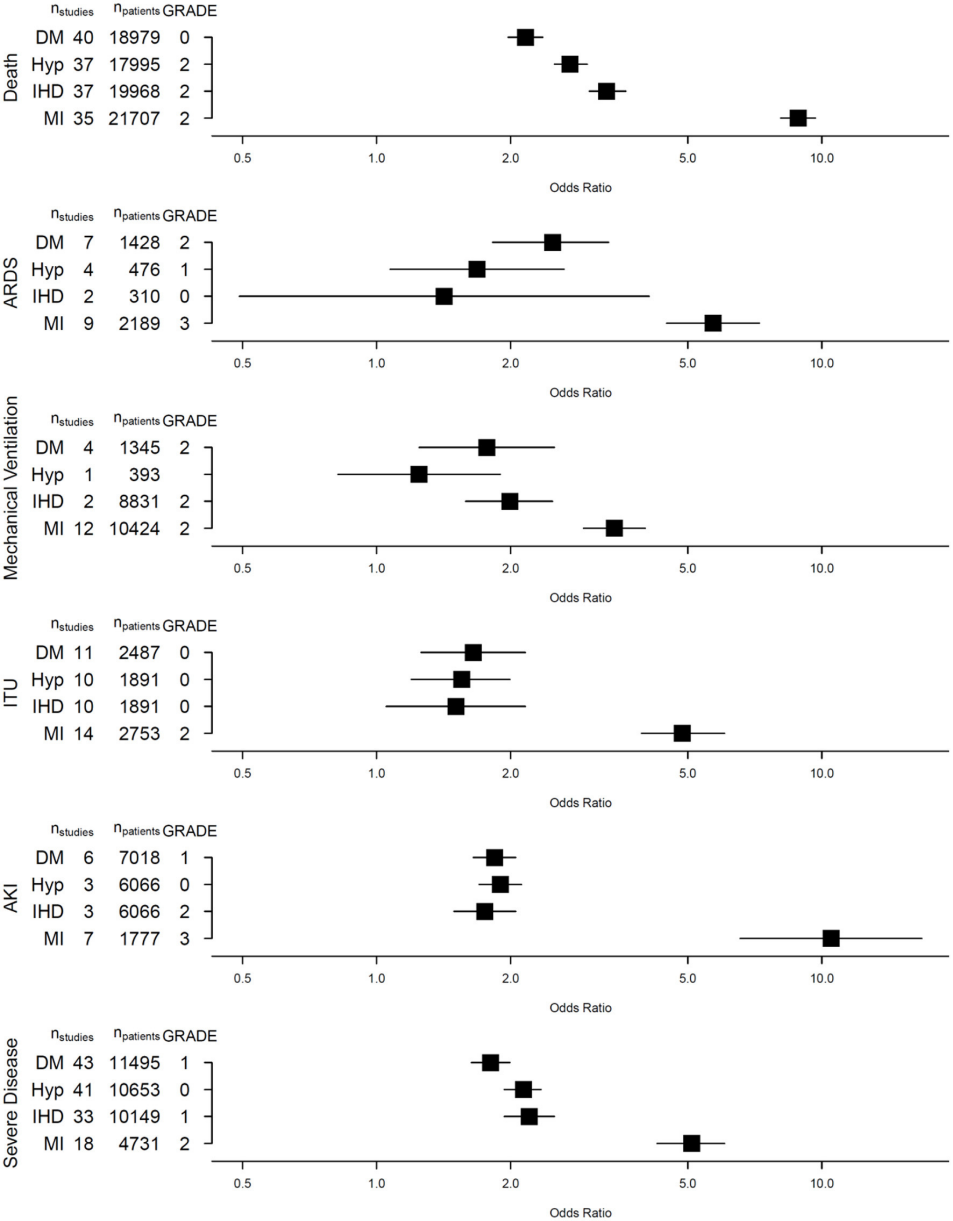
Summary of evidence.

Presence of DM (40 studies, 18,979 patients, 3,791 with DM), HTN (37 studies, 17,995 patients, 6,695 with HTN) or IHD (37 studies, 19,968 patients, 2,619 with IHD) was strongly associated with death from COVID-19 [DM: OR 2.16 (95% confidence interval, CI: 1.97–2.36), HTN: 2.72 (2.51–2.97) and IHD: 3.29 (3.00–3.63), respectively]. These cardiovascular risk factors were also associated with severe COVID-19 disease (Figure 2).

Fewer studies explored the association between DM, HTN and IHD with ARDS, IMV, ITUadm and AKI. Nevertheless, we observed significant associations between these CV risk factors, IHD and ITUadm as well as AKI. While DM and HTN were significant risk factors for developing ARDS, a similar association was not observed in patients with pre-existing IHD [OR 1.42 (0.49–4.10), *p* = 0.30], though only 2 studies involving 310 patients (15 with IHD) were included in this meta-analysis. Only one study (393 patients) explored the association between HTN and IMV, finding no significant association [OR 1.25 (0.82–1.90)].

There was some discrepancy between the fixed-effects and random-effect analysis of the association between IHD and ITUadm. However, the odds ratio and confidence intervals from the fixed-effects model are entirely within the confidence intervals from the random-effect analysis. This would be expected when a random-effect analysis is simply a less efficient estimate of the same parameter or a numerically similar one estimated by the fixed-effects analysis.

### Presence of Myocardial Injury and COVID-19 Outcomes

Forty-nine studies reported on the risks of death and other adverse outcomes associated with the presence of myocardial injury at baseline in patients with COVID-19. Presence of myocardial injury was associated with all six adverse COVID-19 outcomes (Figure 2). There was a near 9-fold increase in the risk of death [OR 8.85; (8.08–9.68)] amongst those with myocardial injury (vs. those without) in 35 studies including 21,707 patients, where 5,225 had myocardial injury and 2,197 patients died. In a meta-analysis of 7 studies comprising 1,777 patients, those with myocardial injury had more than 10-fold increased risk of AKI [OR 10.5; (6.55–16.8)]. Similarly, myocardial injury was also associated with ARDS [OR 5.70; (4.48–7.24)], IMV [3.42; (2.91–4.01)], ITUadm [4.85; (3.93–6.05)] and SCov [OR 5.10 (4.26–6.05)]. Apart from the association between myocardial injury and acute kidney injury, there was substantial heterogeneity in association between studies for all other outcomes (10).

The mean NOS score for all studies combined was 6.95, indicating that they were of satisfactory quality (Appendix D). Moderate to substantial heterogeneity was observed in the majority of meta-analyses. Only four meta-analyses had insignificant heterogeneity (*I*^2^ < 10%), all with small numbers of included studies.

We performed a meta-regression to assess the effect modification by age, gender, publication date and geographic region, on the association of cardiovascular risk factors and myocardial injury on death and severe COVID-19 disease. Overall, advanced age, studies conducted in Asia and male gender showed stronger association between cardiovascular risk factors, ischaemic heart disease and myocardial injury with the COVID-19 outcomes of interest. Gender did not appear to affect the association between myocardial injury and death whereas age did not modify the association between myocardial injury and COVID-19 disease severity. All four modifiers also partly accounted for the observed heterogeneity between studies. Importantly, the GRADE assessment shows low to moderate levels of certainty for associations studied (Appendix E). We did not identify apparent publication bias in our meta-analyses (Appendix F).

## Discussion

The co-existence of HTN, DM, IHD and/or myocardial injury among patients with COVID-19 has been considered to be a harbinger of adverse clinical outcomes. By undertaking this meta-analysis, we confirmed a significant association between four risk factors (HTN, DM, IHD and myocardial injury) and six important adverse COVID-19 outcomes: death, ARDS, IMV, ITUadm, AKI and SCoV. Furthermore, we demonstrated the differential impact of these risk factors on individual COVID-19 outcomes, with myocardial injury emerging as the most adverse indicator of all. These findings may be considered when risk-stratifying unvaccinated patients and unexposed individuals for potential of severe unfavorable outcomes of COVID-19, as well as prioritization of vaccination rollout programmes.

It is worth reviewing the pathogenesis of COVID-19 disease to better understand the detrimental effects of cardiovascular risk factors and myocardial injury on COVID-19 related outcomes. Entry of SARS-CoV-2 into host cells relies on the surface glycoprotein, spike (S) protein, which has a receptor-binding domain (RBD) mediating direct contact with angiotensin-converting enzyme 2 (ACE2). In addition, the betacoronavirus also contains an S1/S2 polybasic cleavage site that is cleaved by cellular transmembrane protease serine 2 (TMPRSS2) and cathepsin L, which further facilitate viral entry. Whilst the predominant tissue tropism of SARS-CoV-2 is that of the alveolar epithelial cells, ACE2 is widely expressed in other organs such as the gastrointestinal tract, myocardium, kidneys and vascular endothelial cells (11). The latter likely contributes to the extrapulmonary manifestations commonly seen in severe COVID-19 disease. Viral replication within alveolar pneumocytes results in the activation of immune cells and release of inflammatory cytokines resulting in a cytokine storm. This is further exacerbated by the downregulation of ACE2 on cell surface membranes, which have a lung-protective and anti-inflammatory effect via the PIP3/Akt signaling pathway. Overall, the propagation of pro-inflammatory cytokine release accelerates clinical deterioration resulting in severe respiratory complications such as ARDS and multiorgan dysfunction (12).

### Cardiovascular Risk Factors, IHD and COVID-19 Outcomes

Pre-existing DM, HTN and IHD were associated with a higher risk of death and severe COVID-19 disease in our meta-analysis, each comprising data from more than 30 studies. These findings concur with pre-existing studies showing worse COVID-19 outcomes in patients with DM and HTN. We further quantified risks of developing specific COVID-19 adverse outcomes, namely death, ARDS, IMV, ITUadm, SCov and AKI in patients with these co-morbidities.

Whilst patients with DM were at an increased risk of developing all six adverse outcomes, the strongest association was seen between DM and the development of ARDS. The pathogenic mechanism underlying severe respiratory complications in patients with DM and COVID-19 is speculated to be due to alveolar-capillary microangiopathy and interstitial fibrosis, resulting from overactive pro-inflammatory pathways and vascular inflammation. A proposed key player to the ongoing inflammation and endothelial damage in DM is interleukin-6 (IL-6), a pro-inflammatory cytokine suggested as a severity predictor of lung disease in DM (13, 14). These pathophysiological changes have previously been associated with obstructive and restrictive lung pathology in patients with DM.

The development of a cytokine storm in patients with severe COVID-19 is well-described (15). Previous studies have found elevated levels of IL-6 in COVID-19 patients, which were independent predictors of COVID-19 disease severity (16). Thus, it is plausible that increased oxidative stress resulting from higher IL-6 levels can lead to rapid progression of microvascular and macrovascular complications in DM patients with pre-existing low-grade vascular inflammation, resulting in increased risk of ARDS and COVID-19 mortality in this cohort (17). This, in part, may account for the observed beneficial effects of IL-6 inhibitor, tocilizumab in hospitalized patients with COVID-19. One could extrapolate the increased odds of IMV and ITUadm in patients with DM and COVID-19 to be due to the requirement for respiratory support in context of ARDS. In addition, multi-organ dysfunction as a consequence of a cytokine storm in DM may contribute further to the need for organ support in intensive care units, especially considering the increased risk for myocardial injury and AKI in patients with DM and SARS-CoV-2 infection. The pre-existing low-grade inflammation coupled with dysregulated immunomodulation in DM patients raises the question of whether a lower threshold or earlier use of IL-6 inhibitors should be considered in this at-risk cohort.

Similarly, patients with pre-existing HTN are at increased odds of COVID-19 related death, SCov and AKI. This may reflect the interlink between SARS-CoV-2 cell entry via angiotensin converting enzyme 2 (ACE2) binding, the renin-angiotensin-aldosterone system (RAAS) and the ubiquity of ACE2 in multiple organs including the lungs, myocardium, kidneys and gastrointestinal tract. Chronic mechanical stress on the vascular wall as a result of increased intraluminal pressure in hypertension leads to endothelial dysfunction, release of reactive oxygen species and a pro-coagulant state. In conjunction with RAAS dysfunction following SARS-CoV-2 infection, this facilitates a pro-inflammatory state, cytokine release syndrome and progression to multi-organ involvement in COVID-19 resulting in more severe disease and adverse outcomes (18).

Whilst also at increased odds of respiratory complications such as ARDS and requirement for IMV, patients with HTN and COVID-19 appear to be at a lower risk of these complications when compared to DM patients. Here, it may be worth raising whether there is a protective role of regular antihypertensives such as ACE-inhibitors and angiotensin receptor blockers (ARB). The reduction in angiotensin-2 levels and suppression of angiotensin-2 binding to angiotensin-I receptors (AT1R) by ACE-inhibitors and ARBs, respectively, may in fact prevent the downstream pro-inflammatory and vasoconstrictive effects of angiotensin-2, lowering the risk of respiratory complications such as ARDS. Indeed, large cohort-based population studies have suggested a protective effect of ACE-inhibitors and ARBs (as compared to calcium channel blockers) against SCoV, death and IMV in patients with hypertension (19).

Another commonly raised question is whether optimal glycaemic control in DM or blood pressure management in HTN have a protective role against adverse COVID-19 outcomes. Certainly, infection with SARS-CoV-2 leads to dysregulated glucose metabolism that can result in elevated IL-6 levels as compared to normoglycaemic patients. Optimal glucose control significantly lowers levels of pro-inflammatory cytokines with improved outcomes in COVID-19 patients with or without diabetes (20). Further studies have also demonstrated adverse effects of hyperglycaemia on COVID-19 outcomes and reduction in the effectiveness of tocilizumab in patients with COVID-19 and hyperglycaemia (21). What remains unclear is whether prior glycaemic control in patients with known diabetes affect outcomes in COVID-19. Population-based studies in the UK have shown an increased risk of COVID-19 mortality with higher glycated hemoglobin (HbA1c) levels (22). This is in contrast to hospital-based cohort studies that have not demonstrated an association between prior glycaemic control with COVID-19-related mortality or invasive mechanical ventilation (23). In our opinion, the increased risk of COVID-19 adverse outcomes in patients with DM are likely reflective of the associated chronic end-organ microvascular and macrovascular complications exacerbated by acute infective sequelae such as increased insulin resistance and an exaggerated inflammatory response. The potential role of antidiabetic medications such as dipeptidylpeptidase-4 (DPP4) inhibitors and glucagon-like peptide 1 (GLP-1) analogs for optimal glycaemic control and their anti-inflammatory properties should be explored in both the acute and chronic stages of COVID-19 infection.

Whilst one may assume patients with poorly controlled blood pressure to be at increased risk of adverse COVID-19 outcomes, this was interestingly not the case in a large observational study of more than 45,000 symptomatic COVID-19 patients with hypertension. Sheppard et al. (24) observed that patients with recent uncontrolled blood pressure had lower odds of COVID-19 related mortality as compared to patients with well-controlled blood pressure. This may suggest a greater role of chronic hypertensive end-organ damage as risk factors for worse COVID-19 outcome.

Importantly, it is worth remembering that many patients have more than one of these studied risk factors. The combined effect of DM and HTN, as well as other components of the “metabolic syndrome” such as dyslipidaemia and obesity is likely greater than its individual components with regards to increased odds of adverse COVID-19 outcome, as demonstrated in specific meta-analyses studying the risk of metabolic syndrome on SCov and death (25). Other comorbidities beyond CV risk factors may play a significant role in adverse COVID-19 outcomes also, as suggested through a prior meta-analysis finding association between cerebrovascular disease and chronic liver disease with IMV in COVID-19 (26).

### Myocardial Injury and COVID-19 Outcomes

Of the four studied risk predictors, myocardial injury had the strongest association with all six adverse COVID-19 outcomes. One could argue this may be a marker of multi-organ involvement and disease severity, rather than a direct pathogenic mechanism. Also, pre-existing cardiovascular diseases such as HTN and IHD increase odds of developing myocardial injury in context of COVID-19 disease (27). Autopsy studies have also suggested an upregulation of ACE2 expression in cardiomyocytes of patients with DM, increasing susceptibility to SARS-CoV-2 entry and myocardial injury in this patient cohort (28). As such, it is not be unreasonable to assume that myocardial injury, as indicated by raised serum troponin levels may simply be a surrogate to the increased odds of adverse COVID-19 outcomes in patients with DM, HTN and IHD. However, Shi et al. (29) and Chen et al. (30) have previously demonstrated that myocardial injury is an independent predictor of mortality in COVID-19. Subsequent multivariable analyses by Wang et al. (31) however showed that this association was only significant on univariate analysis. To date, it remains unclear whether myocardial injury represents a cause or consequence of severe COVID-19 disease.

In our study, we found that 67% (2,197/3,259) of the deceased patients had evidence of myocardial injury, which was associated with a near 9-fold increased odds of COVID-19 related death. Further, our study explores the association between myocardial injury and AKI, demonstrating patients with COVID-19 and myocardial injury are at 10-fold increased odds of developing AKI. 28% of patients with myocardial injury developed AKI as compared to 2.4% of patients without myocardial injury.

The exact mechanism linking myocardial injury with ARDS and AKI in COVID-19 infection is poorly understood. Several hypotheses include a bystander process with cytokine storm and hyperinflammation in severe COVID-19 disease as the driver of multi-organ involvement, endothelial damage and thrombo-inflammation secondary to ACE2-mediated entry of SARS-CoV-2 into endothelial cells of multiple vascular beds and right ventricular dysfunction secondary to increased afterload from raised pulmonary artery pressures in ARDS, thereby causing dysregulated renal blood flow (32, 33).

### Strengths and Limitations

Our meta-analysis pools data from studies across the world and focuses on COVID-19 outcomes in the early phase of the pandemic, before effective evidence-based treatments and vaccines were commonplace. The geographical diversity of included studies overcomes concerns regarding generalisability of earlier meta-analyses, while avoiding biases introduced by novel therapies and vaccines in later stages of the pandemic. Our meta-analysis comprehensively explores the multiple associations between CV risk factors, IHD and myocardial injury with specific COVID-19 outcomes and complications such as AKI and ARDS alongside more commonly reported outcomes such as mortality and IMV.

The generalisability of findings from our meta-analysis was enhanced by not setting language restrictions in our search strategy and by including studies from conventional as well as novel databases (e.g., medRxiv and the WHO COVID-19 database). Moreover, risk of bias was minimized by excluding studies involving specific population cohorts or pre-selected COVID-19 outcomes. By undertaking meta-regression analyses, we were able to explore sources of heterogeneity including age, gender, study recruitment date and geographic region.

Our study has several limitations. Firstly, it is limited in its scope, focusing on outcomes of COVID-19 in a relatively unexposed population in the immediate months following its initial outbreak. Its applicability to populations with high vaccination or herd immunity rates may be limited. Indeed, the emergence of variants with significant mutations affecting virulence features such as transmissibility and pathogenesis may alter aspects of the natural history of COVID-19. At the same time, variants could affect effectiveness of current vaccines (34). In order to delineate the associations between cardiometabolic risk factors and myocardial injury with significant outcomes of COVID-19, we focused on the pre-vaccine phase of the pandemic when rates of adverse outcomes were high and bias arising from access to treatments and vaccines was comparably less pronounced. A necessary trade-off limiting broader applicability to vaccinated populations and potential future variants was accepted.

In addition, the majority of included studies explored adverse outcomes in hospitalized COVID-19 patients. This introduces an inherent selection bias as patients presenting with mild symptoms were likely excluded. Unvaccinated, non-hospitalized individuals with mild COVID-19 symptoms remain an important cohort to study, particularly with the changing trends of disease severity with different SARS-CoV-2 variants.

As our meta-analysis focused specifically on the pre-vaccination phase of the pandemic, we are unable to comment on the association between DM, HTN, IHD and myocardial injury with biomarkers of disease severity including coagulation indicators such as fibrinogen degradation products, prothrombin time, D-dimer and platelets, as these were infrequently reported in earlier studies included. Indeed, the pro-coagulation state in COVID-19 disease is now well-recognized and biomarkers such as D-dimer are used to guide routine use of anticoagulants in COVID-19 patients (35). Whilst findings from the REMAP-CAP trial have not shown a protective effect of antiplatelets in patients with critically-ill patients with COVID-19, it would be interesting to further stratify whether empirical antiplatelet and anticoagulant therapy in patients with different risk profiles (e.g., DM vs. non-DM) will improve overall outcomes (36).

The inclusion of studies conducted at different centers across geographical regions also results in considerable between-study heterogeneity. This could be due to multiple reasons including different sociodemographic factors, varying definitions of outcomes (e.g., ARDS, severe COVID-19 disease) and variations in clinical practice between the countries (we therefore assessed for the effect of geographical variation through meta-regression). For outcomes with fewer studies (ARDS, IMV and AKI), the level of certainty of evidence is low owing to the small study size and wide confidence intervals, limiting the veracity of these findings.

Notwithstanding the above limitations, our meta-analysis quantifies the excess risk of adverse COVID-19 outcomes in unvaccinated patients with pre-existing CV risk factors, IHD and myocardial injury. In addition, we demonstrate the differential impact of these factors on six important adverse COVID-19 outcomes. Our findings will help inform clinicians, policymakers and patients in terms of risk prediction, stratification and resource allocation. Our meta-analysis contributes to the expanding body of evidence reporting on risk factors for poor COVID-19 outcomes, which could inform public health advice regarding social isolation guidelines and vaccine prioritization strategies, especially relevant for LMICs.

Future studies evaluating the altered impact of cardiovascular risk factors such as HTN, DM and IHD on COVID-19 outcomes in a post-vaccination population are required. In addition, the immunogenicity of different COVID-19 vaccines in these patient groups remain poorly elucidated and should be better characterized to guide design of vaccination programmes and choice of vaccine.

## Conclusion

In summary, our meta-analysis demonstrates a significant association between myocardial injury and adverse clinical outcomes in COVID-19 patients. To a lesser extent, we also found that DM, HTN and IHD predict poorer outcomes, especially for death and severe disease. These findings provide comprehensive quantitative data that can be used in risk prediction and risk stratification by clinicians as well as policymakers. It also provides the underpinning evidence for the vaccination policies targeting vulnerable patients.

## Data Availability Statement

The original contributions presented in the study are included in the article/Supplementary Material, further inquiries can be directed to the corresponding author.

## Author Contributions

SN, SK, VK, and AG designed the study. SN, JP, and KM identified studies and extracted data. KR performed the statistical analysis. SN, JP, KM, SK, VK, KR, and AG wrote the manuscript. All authors contributed to the article and approved the submitted version.

## Funding

This work was supported by the British Heart Foundation (BHF) Accelerator Award ‘Cardiac Inflammation' (PI) AA/18/5/34222. Dr. Ajay K Gupta is part-funded by the Barts Charity grant to the Cardiovascular Clinical Trials Unit (CVCTU), William Harvey Research Institute.

## Conflict of Interest

The authors declare that the research was conducted in the absence of any commercial or financial relationships that could be construed as a potential conflict of interest.

## Publisher's Note

All claims expressed in this article are solely those of the authors and do not necessarily represent those of their affiliated organizations, or those of the publisher, the editors and the reviewers. Any product that may be evaluated in this article, or claim that may be made by its manufacturer, is not guaranteed or endorsed by the publisher.

## References

[1] Ritchie H Mathieu E Rodés-Guirao L Appel C Giattino C Ortiz-Ospina E . Coronavirus Pandemic (COVID-19). Our World in Data (2020) Mar 5. Available online at: https://ourworldindata.org/ (accessed March 27, 2022).

[2] Gyawali N Al-Amin HM. Living and dying with COVID-19 in South Asian low- and middle-income countries. Front Public Health. (2021) 9:600878. 10.3389/fpubh.2021.600878 34277531 PMC8281030

[3] Biccard BM Gopalan PD Miller M Michell WL Thomson D Ademuyiwa A . Patient care and clinical outcomes for patients with COVID-19 infection admitted to African high-care or intensive care units (ACCCOS): a multicentre, prospective, observational cohort study. Lancet. (2021) 397:1885–94. 10.1016/S0140-6736(21)00441-4 34022988 PMC8137309

[4] Danza P. SARS-CoV-2 Infection and Hospitalization Among Adults Aged ≥18 Years, by Vaccination Status, Before and During SARS-CoV-2 B.1.1.529 (Omicron) Variant Predominance — Los Angeles County, California, November 7, 2021–January 8, 2022. MMWR Morb Mortal Wkly Rep. (2022) 71:177–81. 10.15585/mmwr.mm7105e1 35113851 PMC8812833

[5] Hu H Yao N Qiu Y. Comparing rapid scoring systems in mortality prediction of critically ill patients with novel coronavirus disease. Acad Emerg Med. (2020) 27:461–8. 10.1111/acem.13992 32311790 PMC7264631

[6] Du R-H Liu L-M Yin W Wang W Guan L-L Yuan M-L . Hospitalization and critical care of 109 decedents with COVID-19 Pneumonia in Wuhan, China. Ann Am Thorac Soc. (2020) 17:839–46. 10.1513/AnnalsATS.202003-225OC 32255382 PMC7328178

[7] Lin DY Zeng D. On the relative efficiency of using summary statistics versus individual-level data in meta-analysis. Biometrika. (2010) 97:321–32. 10.1093/biomet/asq006 23049122 PMC3412575

[8] Higgins JPT Thompson SG Deeks JJ Altman DG. Measuring inconsistency in meta-analyses. BMJ. (2003) 327:557–60. 10.1136/bmj.327.7414.557 12958120 PMC192859

[9] Qijun Li K Rice K. Improved inference for fixed-effects meta-analysis of 2 × 2 tables. Res Synth Methods. (2020) 11:387–96. 10.1002/jrsm.1401 32092228

[10] Cochrane Handbook for Systematic Reviews of Interventions. Version 5.1.0. Available online at: https://handbook-5-1.cochrane.org/chapter_9/9_5_2_identifying_and_measuring_heterogeneity.htm - Google Search (accessed March 27, 2022).

[11] Harrison AG Lin T Wang P. Mechanisms of SARS-CoV-2 transmission and pathogenesis. Trend Immunol. (2020) 41:1100–15. 10.1016/j.it.2020.10.004 33132005 PMC7556779

[12] Chen R Lan Z Ye J Pang L Liu Y Wu W . cytokine storm: the primary determinant for the pathophysiological evolution of COVID-19 deterioration. Front Immunol. (2021) 12:589095. 10.3389/fimmu.2021.589095 33995341 PMC8115911

[13] Sardu C Gargiulo G Esposito G Paolisso G Marfella R. Impact of diabetes mellitus on clinical outcomes in patients affected by COVID-19. Cardiovasc Diabetol. (2020) 19:76. 10.1186/s12933-020-01047-y 32527257 PMC7289072

[14] Khateeb J Fuchs E Khamaisi M. Diabetes and lung disease: a neglected relationship. Rev Diabet Stud. (2019) 15:1–15. 10.1900/RDS.2019.15.1 30489598 PMC6760893

[15] Ragab D Salah Eldin H Taeimah M Khattab R Salem R. The COVID-19 cytokine storm; what we know so far. Front Immunol. (2020) 11:1446. 10.3389/fimmu.2020.01446 32612617 PMC7308649

[16] Zeng Z Yu H Chen H Qi W Chen L Chen G . Longitudinal changes of inflammatory parameters and their correlation with disease severity and outcomes in patients with COVID-19 from Wuhan, China. Crit Care. (2020) 24:525. 10.1186/s13054-020-03255-0 32854750 PMC7450961

[17] Lim S Bae JH Kwon H-S Nauck MA. COVID-19 and diabetes mellitus: from pathophysiology to clinical management. Nat Rev Endocrinol. (2021) 17:11–30. 10.1038/s41574-020-00435-4 33188364 PMC7664589

[18] Muhamad S-A Ugusman A Kumar J Skiba D Hamid AA Aminuddin A. COVID-19 and hypertension: the what, the why, and the how. Front Physiol. (2021) 12:665064. 10.3389/fphys.2021.665064 34012410 PMC8126692

[19] Semenzato L Botton J Drouin J Baricault B Vabre C Cuenot F . Antihypertensive drugs and COVID-19 risk. Hypertension. (2021) 77:833–42. 10.1161/HYPERTENSIONAHA.120.16314 33423528 PMC7884243

[20] Sardu C D'Onofrio N Balestrieri ML Barbieri M Rizzo MR Messina V . Outcomes in patients with hyperglycemia affected by COVID-19: can we do more on glycemic control? Diabetes Care. (2020) 43:1408–15. 10.2337/dc20-0723 32430456 PMC7305003

[21] Marfella R Paolisso P Sardu C Bergamaschi L D'Angelo EC Barbieri M . Negative impact of hyperglycaemia on tocilizumab therapy in COVID-19 patients. Diabetes Metab. (2020) 46:403–5. 10.1016/j.diabet.2020.05.005 32447102 PMC7241396

[22] Holman N Knighton P Kar P O'Keefe J Curley M Weaver A . Risk factors for COVID-19-related mortality in people with type 1 and type 2 diabetes in England: a population-based cohort study. Lancet Diabetes Endocrinol. (2020) 8:823–33. 10.1016/S2213-8587(20)30271-0 32798471 PMC7426091

[23] Bloomgarden Z. Does glycemic control affect outcome of COVID-19? J Diabetes. (2020) 12:868-9. 10.1111/1753-0407.13116 32969135 PMC7537073

[24] Sheppard JP Nicholson BD Lee J McGagh D Sherlock J Koshiaris C . Association between blood pressure control and coronavirus disease 2019 outcomes in 45 418 symptomatic patients with hypertension. Hypertension. (2021) 77:846–55. 10.1161/HYPERTENSIONAHA.120.16472 33325240 PMC7884248

[25] Rico-Martín S Calderón-García JF Basilio-Fernández B Clavijo-Chamorro MZ Sánchez Muñoz-Torrero JF. Metabolic syndrome and its components in patients with COVID-19: severe acute respiratory syndrome (SARS) and mortality. a systematic review and meta-analysis. J Cardiovasc Dev Dis. (2021) 8:162. 10.3390/jcdd8120162 34940517 PMC8708678

[26] Patel U Malik P Usman MS Mehta D Sharma A Malik FA . Age-adjusted risk factors associated with mortality and mechanical ventilation utilization amongst COVID-19 hospitalizations-a systematic review and meta-analysis. SN Compr Clin Med. (2020) 2:1740–9. 10.1007/s42399-020-00476-w 32904541 PMC7456201

[27] Fan Q Zhu H Zhao J Zhuang L Zhang H Xie H . Risk factors for myocardial injury in patients with coronavirus disease 2019 in China. ESC Heart Failure. (2020) 7:4108–17. 10.1002/ehf2.13022 33006440 PMC7537185

[28] D'Onofrio N Scisciola L Sardu C Trotta MC De Feo M Maiello C . Glycated ACE2 receptor in diabetes: open door for SARS-CoV-2 entry in cardiomyocyte. Cardiovasc Diabetol. (2021) 20:99. 10.1186/s12933-021-01286-7 33962629 PMC8104461

[29] Shi S Qin M Shen B Cai Y Liu T Yang F . Association of cardiac injury with mortality in hospitalized patients with COVID-19 in Wuhan, China. JAMA Cardiol. (2020) 5:802–10. 10.1001/jamacardio.2020.0950 32211816 PMC7097841

[30] Chen C Yan JT Zhou N Zhao JP Wang DW. [Analysis of myocardial injury in patients with COVID-19 and association between concomitant cardiovascular diseases and severity of COVID-19]. Zhonghua Xin Xue Guan Bing Za Zhi. (2020) 48:567-71. 10.3760/cma.j.cn112148-20200225-00123 32141280

[31] Wang L He W Yu X Hu D Bao M Liu H . Coronavirus disease 2019 in elderly patients: characteristics and prognostic factors based on 4-week follow-up. J Infect. (2020) 80:639–45. 10.1016/j.jinf.2020.03.019 32240670 PMC7118526

[32] Gupta A Madhavan MV Sehgal K Nair N Mahajan S Sehrawat TS . Extrapulmonary manifestations of COVID-19. Nat Med. (2020) 26:1017–32. 10.1038/s41591-020-0968-3 32651579 PMC11972613

[33] Varga Z Flammer AJ Steiger P Haberecker M Andermatt R Zinkernagel AS . Endothelial cell infection and endotheliitis in COVID-19. Lancet. (2020) 395:1417–8. 10.1016/S0140-6736(20)30937-5 32325026 PMC7172722

[34] Saxena SK Kumar S Ansari S Paweska JT Maurya VK Tripathi AK . Transmission dynamics and mutational prevalence of the novel severe acute respiratory syndrome coronavirus-2 Omicron variant of concern. J Med Virol. (2022) 94:2160–6. 10.1002/jmv.27611 35050521 PMC9015611

[35] Sardu C Gambardella J Morelli MB Wang X Marfella R Santulli G. Hypertension, thrombosis, kidney failure, and diabetes: is COVID-19 an endothelial disease? a comprehensive evaluation of clinical and basic evidence. J Clin Med. (2020) 9:1417. 10.3390/jcm9051417 32403217 PMC7290769

[36] REMAP-CAP Writing Committee for the REMAP-CAP Investigators Bradbury CA Lawler PR Stanworth SJ McVerry BJ McQuilten Z. Effect of antiplatelet therapy on survival and organ support-free days in critically Ill patients with COVID-19: a randomized clinical trial. JAMA. (2022) 327:1247–59. 10.1001/jama.2022.2910 35315874 PMC8941448

