# Supplementary material for: Quantifying the Excess Risk of Adverse COVID-19 Outcomes in Unvaccinated Individuals With Diabetes Mellitus, Hypertension, Ischaemic Heart Disease or Myocardial Injury: A Meta-Analysis
Source: Front Cardiovasc Med. 2022 Apr 26;9:871151. doi: 10.3389/fcvm.2022.871151 (PMC9090337; doi:10.3389/fcvm.2022.871151)
Supplement: Supplementary file 1 [file Data_Sheet_1.pdf]

Quantifying the excess risk of adverse Covid-19 outcomes in unvaccinated individuals with Diabetes Mellitus, Hypertension, Ischaemic Heart Disease or Myocardial Injury: a Meta-Analysis.

Supplementary Materials

Appendix A: search strategy

Evidence search: impact of cardiac injury on COVID-19. Adam Tocock. (30th May 2020 and 16th July 2020). LONDON, UK: Barts Health Knowledge and Library Services.

Medline

Number of results
Searched on 30th May 2020: 464
Searched on 16th July 2020: 983
Results from May 2020: 196
Total excluding duplicate results from May 2020 = 464 + 983 – 196 = 1251

Strategy 16th July 2020 (initial search on 30th May 2020 used identical criteria except date limits)
1 Medline (coronavir* OR "Corona vir*" OR ncov* OR "n-cov*" OR COVID*).ti,ab 42477
2 Medline ("2019-nCoV" OR "SARS-CoV*" OR "MERS-CoV*" OR "Severe Acute Respiratory Syndrome" OR "Middle East Respiratory Syndrome").ti,ab 11343
3 Medline exp CORONAVIRUS/ OR exp "CORONAVIRUS INFECTIONS"/ 26327
4 Medline (1 OR 2 OR 3) 52484
5 Medline ((heart* OR cardiac OR cardiovascula*) ADJ3 (injur* OR damag* OR fail* OR infarct* OR attack*)).ti,ab 230909
6 Medline (troponin OR myocardi* OR arrhythmi*).ti,ab 455479
7 Medline exp "HEART DISEASES"/ 1122464
8 Medline exp TROPONIN/ 17247
9 Medline ("CK" OR CKMB OR "CK MB" OR "creatine kinase").ti,ab 37325
10 Medline exp "CREATINE KINASE"/ 26134
11 Medline (nstemi OR stemi OR ACS OR "acute coronary syndr*").ti,ab 50254
12 Medline exp "MYOCARDIAL ISCHEMIA"/ 428159
13 Medline (5 OR 6 OR 7 OR 8 OR 9 OR 10 OR 11 OR 12) 1372379
14 Medline (4 AND 13) 1193
15 Medline (4 AND 13) [DT FROM 2019] 983

CINAHL

Number of results

Searched on 30th May 2020: 42
Searched on 16th July 2020: 191
Results from May 2020: 48
Total excluding duplicate results from May 2020 = 42 + 191 - 48 = 185

Strategy 16th July 2020 (initial search on 30th May 2020 used identical criteria except date limits)

16 CINAHL (coronavir* OR "Corona vir*" OR ncov* OR "n-cov*" OR COVID*).ti,ab 4679
17 CINAHL ("2019-nCoV" OR "SARS-CoV*" OR "MERS-CoV*" OR "Severe Acute Respiratory Syndrome" OR "Middle East Respiratory Syndrome").ti,ab 2019
18 CINAHL exp CORONAVIRUS/ OR exp "CORONAVIRUS INFECTIONS"/ 5604
19 CINAHL (16 OR 17 OR 18) 7264
20 CINAHL ((heart* OR cardiac OR cardiovascula*) ADJ3 (injur* OR damag* OR fail* OR infarct* OR attack*)).ti,ab 68285
21 CINAHL (troponin OR myocardi* OR arrhythmi*).ti,ab 93054
22 CINAHL exp "HEART DISEASES"/ 287772
23 CINAHL exp TROPONIN/ 5105
24 CINAHL ("CK" OR CKMB OR "CK MB" OR "creatine kinase").ti,ab 4872
25 CINAHL exp "CREATINE KINASE"/ 4125
26 CINAHL (nstemi OR stemi OR ACS OR "acute coronary syndr*").ti,ab 17672
27 CINAHL exp "MYOCARDIAL ISCHEMIA"/ 104434
28 CINAHL (20 OR 21 OR 22 OR 23 OR 24 OR 25 OR 26 OR 27) 344780
29 CINAHL (19 AND 28) 236
30 CINAHL (19 AND 28) [DT FROM 2019] 191

Embase

Number of results
Searched on 30th May 2020: 498
Searched on 16th July 2020: 1036
Results from May 2020: 142
Total excluding duplicate results from May 2020 = 498 + 1036 – 142 = 1392

Strategy 16th July 2020 (initial search on 30th May 2020 used identical criteria except date limits)

31 EMBASE (coronavir* OR "Corona vir*" OR ncov* OR "n-cov*" OR COVID*).ti,ab 29718
32 EMBASE ("2019-nCoV" OR "SARS-CoV*" OR "MERS-CoV*" OR "Severe Acute Respiratory Syndrome" OR "Middle East Respiratory Syndrome").ti,ab 15887
33 EMBASE exp *CORONAVIRINAE/ 8329
34 EMBASE (31 OR 32 OR 33) 49078
35 EMBASE ((heart* OR cardiac OR cardiovascula*) ADJ3 (injur* OR damag* OR fail* OR infarct* OR attack*)).ti,ab 351348
36 EMBASE (troponin OR myocardi* OR arrhythmi*).ti,ab 640984
37 EMBASE exp *"HEART DISEASE"/ 1020288
38 EMBASE exp *TROPONIN/ 15436
39 EMBASE ("CK" OR CKMB OR "CK MB" OR "creatine kinase").ti,ab 53356
40 EMBASE exp *"CREATINE KINASE"/ 9954
41 EMBASE (nstemi OR stemi OR ACS OR "acute coronary syndr*").ti,ab 95096
42 EMBASE (35 OR 36 OR 37 OR 38 OR 39 OR 40 OR 41) 1468313
43 EMBASE (34 AND 42) 1265
44 EMBASE (34 AND 42) [DT FROM 2019] 1036

EMCARE
Number of results
Searched on 30th May 2020: 43
Searched on 16th July 2020: 120
Results from May 2020: 23
Total excluding duplicate results from May 2020 = 43 + 120 – 23 = 140

Strategy 16th July 2020 (initial search on 30th May 2020 used identical criteria except date limits)
45 EMCARE (coronavir* OR "Corona vir*" OR ncov* OR "n-cov*" OR COVID*).ti,ab 7475
46 EMCARE ("2019-nCoV" OR "SARS-CoV*" OR "MERS-CoV*" OR "Severe Acute Respiratory Syndrome" OR "Middle East Respiratory Syndrome").ti,ab 2160
47 EMCARE exp *CORONAVIRINAE/ 914
48 EMCARE (45 OR 46 OR 47) 4657
49 EMCARE ((heart* OR cardiac OR cardiovascula*) ADJ3 (injur* OR damag* OR fail* OR infarct* OR attack*)).ti,ab 70492
50 EMCARE (troponin OR myocardi* OR arrhythmi*).ti,ab 117579
51 EMCARE exp *"HEART DISEASE"/ 201385
52 EMCARE exp *TROPONIN/ 3190
53 EMCARE ("CK" OR CKMB OR "CK MB" OR "creatine kinase").ti,ab 6956
54 EMCARE exp *"CREATINE KINASE"/ 936
55 EMCARE (nstemi OR stemi OR ACS OR "acute coronary syndr*").ti,ab 18512
56 EMCARE (49 OR 50 OR 51 OR 52 OR 53 OR 54 OR 55) 282045
57 EMCARE (48 AND 56) 152
58 EMCARE (48 AND 56) [DT FROM 2019] 120

BNI
Number of results
Searched on 30th May 2020: 16
Searched on 16th July 2020: 28
Results from May 2020: 13
Total excluding duplicate results from May 2020 = 43 + 120 – 23 = 31

Strategy 16th July 2020 (initial search on 30th May 2020 used identical criteria except date limits)
59 BNI (coronavir* OR "Corona vir*" OR ncov* OR "n-cov*" OR COVID*).ti,ab 842
60 BNI ("2019-nCoV" OR "SARS-CoV*" OR "MERS-CoV*" OR "Severe Acute Respiratory Syndrome" OR "Middle East Respiratory Syndrome").ti,ab 566
61 BNI "COVID-19"/ 1032
62 BNI (59 OR 60 OR 61) 1689
63 BNI ((heart* OR cardiac OR cardiovascula*) ADJ3 (injur* OR damag* OR fail* OR infarct* OR attack*)).ti,ab 8256
64 BNI "CARDIOVASCULAR DISEASE"/ 19763
65 BNI (troponin OR myocardi* OR arrhythmi*).ti,ab 7171
66 BNI ("CK" OR CKMB OR "CK MB" OR "creatine kinase").ti,ab 184
67 BNI (nstemi OR stemi OR ACS OR "acute coronary syndr*").ti,ab 1406
68 BNI (63 OR 64 OR 65 OR 66 OR 67) 29179
69 BNI (62 AND 68) 35
70 BNI (62 AND 68) [DT FROM 2019] 28

Results from WHO's COVID-19 database:

Number of results
Searched on 16th July 2020, date limited to results from the year 2020.
1195 results were found with this search.
Although an initial search was done on 30th May 2020; the second search entirely replaced the initial search as the date limits were both ‘2020’.

Strategy:
tw:((tw:((heart* OR cardiac OR cardiovascula*) AND (injur* OR damag* OR fail* OR infarct* OR attack*))) OR (tw:(troponin OR myocardi* OR arrhythmi* OR "CK" OR ckmb OR "CK MB" OR "creatine kinase")) OR (tw:(nstemi OR stemi OR acs OR "acute coronary syndra" OR "acute coronary syndrome")))

https://search.bvsalud.org/global-literature-on-novel-coronavirus-2019-ncov/?output=site&lang=en&from=0&sort=DATENTRY_DESC&format=summary&count=100&fb=&page=1&skfp=&index=tw&q=%28tw%3A%28%28heart*+OR+cardiac+OR+cardiovascula*%29+AND+%28injur*+OR+damag*+OR+fail*+OR+infarct*+OR+attack*%29%29%29+OR+%28tw%3A%28troponin+OR+myocardi*+OR+arrhythmi*+or+%22CK%22+OR+CKMB+OR+%22CK+MB%22+OR+%22creatine+kinase%22%29%29+OR+%28tw%3A%28nstemi+OR+stemi+OR+ACS+OR+%22acute+coronary+syndra%22+OR+%22acute+coronary+syndrome%22%29%29

Results from search on MedRxiv:

Number of results
Searched on 30th May 2020: 115
Searched on 16th July 2020: 5 (date limited to articles posted between 31 May 2020 and 16 July 2020)
Total results: 120

Strategy:
title "covid* corona* ncov* mers sars" (match any words) and abstract or title "cardiac injury" (match phrase words)"

Appendix B: Included studies

Author	Journal	Date of publication	Country	N	Age	Male	Co-morbidities included	Outcomes studied	NOS	Ref
Franks C	J Appl Lab Med	30/07/2020	USA	182	60.3	103	Trop	Death, ARDS, I+V, AKI	★★★★★★★	[1]
Deng Q	Int J Card	15/07/2020	China	112	61.6	57	HTN, DM, IHD, Trop	Death, I+V, ITU, Severity	★★★★★	[2]
Chen X	J Clin Lab Ana	12/07/2020	China	73	65.8	42	HTN, DM, IHD	Death, I+V	★★★★★★★	[3]
Shang J	Am J Med	10/07/2020	China	584	53.0	277	HTN, DM, IHD, Trop	Death, ARDS, I+V, ITU, AKI, Severity	★★★★★★★★	[4]
Aloisio E	Arch Path Lab Med	10/07/2020	Italy	427	61.3	293	HTN, DM, IHD	Death, ITU	★★★★★★★★	[5]
Ferrante F	Cardiovasc Res	08/07/2020	Italy	332	65.9	237	HTN, DM, IHD, Trop	Death, I+V, ITU	★★★★★★★	[6]
Yang X	medRxiv	07/07/2020	China	619	58.4	297	HTN, DM, IHD, Trop	Death	★★★★★★★★	[7]
Ni W	JACC	07/07/2020	China	176	65.7	101	HTN, DM, IHD	Death	★★★★★★★★	[8]
Liu D	Clin Infect Prac	02/07/2020	China	214	48.7	114	HTN, DM, IHD, Trop	I+V, Severity	★★★★★★	[9]
van den Heuvel F	Neth Heart J	01/07/2020	Netherlands	51	60.7	41	HTN, DM, IHD, Trop	Death, I+V, ITU, AKI, Severity	★★★★★★	[10]
Pelayo J	Cardiorenal Med	01/07/2020	USA	223	65.9	115	HTN, DM, IHD	Death, I+V, AKI	★★★★★★★★	[11]
Lodigiani C	Thromb Res	01/07/2020	Italy	388	63.3	264	HTN, DM, IHD	Death, ITU	★★★★★★	[12]
Sun L	J Clin Virol	01/07/2020	China	336	45.0	177	HTN, DM, IHD	Severity	★★★★★★★★★	[13]
McCullough S	J Card Fail	01/07/2020	USA	756	63.5	478	HTN, DM, IHD	Death	★★★★★★★	[14]
Zhao Y	J Med Virol	26/06/2020	China	75	51.7	41	HTN, DM	Severity	★★★★★★	[15]
Bonetti G	Clin Lab Chem Med	25/06/2020	Italy	144	N/A	96	DM, IHD	Death	★★★★★★★	[16]
Aggarwal AS	J Assoc Phys India	23/06/2020	India	32	53.6	19	HTN, DM, IHD	Severity	★★★★★★★★	[17]

Iaccarino D	Hypertension	22/06/2020	Italy	1591	66.5	1018	HTN, DM, IHD	Death	★★★★★★★★	18
Barman HA	Coron Art Dis	19/06/2020	Turkey	607	62.5	337	HTN, DM, IHD, Trop	Death, ARDS, ITU, AKI, Severity	★★★★★★★★	19
Lorente RA	Cardiol J	19/06/2020	Spain	224	66.6	95	HTN, DM, IHD, Trop	Death, ARDS, ITU	★★★★★★★★★	20
Cao Z	PLOS one	17/06/2020	China	80	53.0	38	HTN, DM, IHD, Trop	Death, ITU, Severity	★★★★★★★★	21
Suleyman F	JAMA Netw Open	16/06/2020	USA	355	61.4	165	HTN, DM, IHD, Trop	Death, ARDS, I+V, ITU, AKI,	★★★★★★★★★	22
Mani V	J Med Int Res	16/06/2020	USA	184	64.7	111	HTN, DM, IHD	Death, I+V	★★★★★★★★	23
Jang J	J Korean Med Sci	15/06/2020	South Korea	110	56.9	48	HTN, DM, IHD	Death, Severity	★★★★★★★★	24
Nie S	Circulation	15/06/2020	China	311	62.3	190	Trop	Death, Severity	★★★★★★★★	25
Xie Y	Circ J	13/06/2020	China	62	64.1	27	HTN, DM, IHD	Death, ITU, Severity	★★★★★★★★	26
Ciceri F	Clin Immunol	12/06/2020	Italy	410	65.3	299	HTN, DM, IHD	Death, ITU	★★★★★★★★	27
Goyal P	N Eng J Med	11/06/2020	USA	393	61.5	238	HTN, DM, IHD	Death, I+V, AKI	★★★★★★	28
Mikami T	J Gen Intern Med	11/06/2020	USA	6493	58.0	3538	HTN, DM, Trop	Death	★★★★★★★★★	29
Okoh S	Int J Equity Health	10/06/2020	USA	251	61.7	N/A	HTN, DM, IHD	Death	★★★★★★★★	30
Violi F	Antiox and Redox Signal	08/06/2020	Italy	319	68.2	193	HTN, DM, IHD	Death, ITU	★★★★★	31
Deng Y	Chin Med J	05/06/2020	China	225	54.0	124	HTN, DM, IHD	Death, ARDS, I+V, AKI	★★★★★★★	32
Feng Y	Am J Resp Crit Care Med	01/06/2020	China	476	52.3	271	HTN, DM, IHD, Trop	Death, I+V, Severity	★★★★★★	33
Yang AP	Aging	01/06/2020	China	93	46.4	56	HTN, DM, IHD	Severity	★★★★★★	34
Zengin A	J Surg Med	30/05/2020	Turkey	49	56.2	29	HTN, DM, IHD, Trop	Death, ITU	★★★★★★	35
Wang CZ	J Med Virol	29/05/2020	China	85	59.4	45	HTN, DM, IHD	ITU, Severity	★★★★★★	36
Rath D	Clin Res Cardiol	28/05/2020	Germany	123	68.0	77	HTN, DM, IHD	Death, I+V, ITU, AKI	★★★★★★★	37

Garibaldi B	medRxiv	26/05/2020	USA	832	62.3	443	HTN, DM, IHD, Trop	Death, I+V, Severity	★★★★★★★★	38
Yang Q	J Clin Pharm Ther	25/05/2020	China	136	54.7	66	HTN, DM, IHD, Trop	Death, ARDS, AKI, Severity	★★★★★★	39
Petrilli C	BMJ	22/05/2020	USA	2729	62.7	1672	HTN, DM, IHD	Death, Severity	★★★★★★★★★	40
Chu S	Diab Metab J	21/05/2020	South Korea	*110	*56.9	*48	HTN, DM, IHD, Trop	Death, ARDS, I+V, ITU, AKI,	★★★★★★★★	41
Zhang Y	Diab Res Clin Prac	21/05/2020	China	258	63.3	138	HTN, DM, IHD	Death, ARDS, I+V, AKI, Severity	★★★★★★	42
Schiller M	J Comm Hosp Int Med Pers	21/05/2020	Germany	42	71.3	21	HTN, DM, IHD	Death, ITU	★★★★★★	43
Lu Y	medRxiv	16/05/2020	China	121	57.3	66	HTN, DM, IHD, Trop	Death, ARDS, I+V, AKI, Severity	★★★★★★★	44
Hirsch J	Kidney Int	16/05/2020	USA	5449	63.7	3317	HTN, DM, IHD	Death, I+V, ITU, AKI	★★★★★★★★	45
Kuno T	Am Heart J	15/05/2020	USA	8438	57.7	4544	HTN, DM, IHD, Trop	Death, I+V	★★★★★★★	46
Zhang J	medRxiv	09/05/2020	China	135	55.3	67	HTN, DM, IHD, Trop	Death, ARDS, ITU, AKI, Severity	★★★★★★	47
Inciardi R	Eur Heart J	08/05/2020	Italy	99	67.0	80	HTN, DM, IHD	Death, ARDS, I+V, ITU	★★★★★★★	48
Borobia A	medRxiv	06/05/2020	Spain	2226	61.7	1074	HTN, DM, IHD, Trop	Death, ARDS, I+V, ITU, AKI, Severity	★★★★★★	49
DU RH	Eur Resp J	05/05/2020	China	179	57.6	997	HTN, DM, IHD	Death	★★★★★★★★	50
Hu L	Clin Infect Dis	03/05/2020	China	323	58.3	166	HTN, DM, IHD, Trop	Death, ARDS, I+V, AKI, Severity	★★★★★★★★	51
Wang K	Clin Infect Dis	03/05/2020	China	296	47.3	140	HTN, DM, IHD	Death	★★★★★★★★	52
Guan WJ	N Engl J Med	30/04/2020	China	1099	46.7	637	HTN, DM, IHD	Death, ARDS, I+V, ITU, AKI, Severity	★★★★★★	53
Wei JF	Heart	30/04/2020	China	101	48.3	54	HTN, DM, IHD, Trop	Death, I+V, ITU, Severity	★★★★★★★	54
Wang D	Crit Care	30/04/2020	China	107	50.7	57	HTN, DM, IHD, Trop	Death, ARDS, I+V, AKI	★★★★★★★★★	55
Feng X	medRxiv	29/04/2020	China	114	64.0	71	HTN, DM, IHD, Trop	Death, ARDS, I+V, ITU, AKI, Severity	★★★★★★★★	56

Zhao X	BMC Infect Dis	29/04/2020	China	91	15.3	49	HTN, DM, Trop	Death, I+V, AKI, Severity	★★★★★★	57
Zhang J	Chin Crit Care Med	28/04/2020	China	394	55.0	186	HTN, DM, IHD	AKI	★★★★★★	58
Tomlins J	J Infect	27/04/2020	United Kingdom	95	72.0	60	HTN, DM, IHD	Death	★★★★★★	59
Hong KS	YMJ	24/04/2020	South Korea	*98	*55.4	*38	HTN, DM, IHD, Trop	Death, ARDS, I+V, ITU, AKI,	★★★★★★	60
Zhu Z	Int J Infect Dis	22/04/2020	China	127	50.9	45	HTN, DM, IHD	Severity	★★★★★★★★	61
Liu J	EBioMedicine	18/04/2020	China	40	48.7	15	HTN, DM	Death, ARDS, Severity	★★★★★★	62
Chen X	Clin Infect Dis	17/04/2020	China	48	64.6	37	HTN, DM, IHD	Death, Severity	★★★★★★★	63
Wang L	Chin J Cardiovasc Dis	14/04/2020	China	202	61.3	88	HTN, DM, IHD	Death, Severity	★★★★★★★★	64
Li X	J Allerg Clin Immunol	12/04/2020	China	548	59.0	279	HTN, DM, IHD, Trop	Death, ARDS, I+V, AKI, Severity	★★★★★★★★	65
He R	J Clin Virol	12/04/2020	China	204	48.3	79	HTN, DM, IHD	Death, Severity	★★★★★★	66
Fang X	J Infect	11/04/2020	China	78	45.5	44	HTN, DM, IHD	ARDS, Severity	★★★★★★	67
Mao L	JAMA Neurol	10/04/2020	China	214	52.7	87	HTN, DM, IHD	Death, Severity	★★★★★★	68
Gu T	medRxiv	03/04/2020	China	275	66.4	173	HTN, DM, IHD	Death	★★★★★★★★	69
Dreher M	Dtsch Arztebl Int	01/04/2020	Germany	50	66.3	33	HTN, DM	Death, ARDS, I+V, ITU	★★★★★★	70
Wang L	Am J Nephrol	31/03/2020	China	116	53.7	67	HTN, DM	Death, ARDS, Severity	★★★★★★	71
Han H	J Med Virol	31/03/2020	China	273	58.9	97	Trop	Death, Severity	★★★★★★	72
Fan L	medRxiv	30/03/2020	China	55	46.8	30	HTN, DM, IHD	Death, ARDS, Severity	★★★★★★★	73
Li YK	Curr Med Sci	30/03/2020	China	25	51.0	12	HTN, DM, IHD, Trop	Death, Severity	★★★★★★	74
Chen G	J Clin Invest	27/03/2020	China	21	57.0	17	HTN, DM	Death, ARDS, I+V, AKI, Severity	★★★★★★★	75
Guo T	JAMA Cardiol	27/03/2020	China	187	58.5	91	HTN, DM, IHD, Trop	Death, ARDS, I+V, AKI	★★★★★★★★	76

Li K	medRxiv	27/03/2020	China	128	57.3	59	HTN, DM, IHD, Trop	Death	★★★★★★★★★	[77]
Zhou Y	medRxiv	27/03/2020	China	377	53.7	170	HTN, DM, IHD	Severity	★★★★★★★	[78]
Han Y	medRxiv	27/03/2020	China	47	61.0	26	HTN, DM, IHD	Severity	★★★★★★★	[79]
Shi S	JAMA Cardiol	25/03/2020	China	416	60.0	205	HTN, DM, IHD, Trop	Death, ARDS, I+V, AKI	★★★★★★★★	[80]
Zhang	medRxiv	24/03/2020	China	48	70.6	33	HTN, DM, IHD, Trop	Death, I+V, Severity	★★★★★★★	[81]
Luo X	medRxiv	23/03/2020	China	403	54.3	193	HTN, DM, IHD, Trop	Death, ARDS, I+V, ITU, AKI, Severity	★★★★★★★	[82]
Yan S	medRxiv	23/03/2020	China	168	49.7	81	HTN, DM, IHD, Trop	Death, ARDS, I+V, AKI, Severity	★★★★★★	[83]
Ma KL	medRxiv	23/03/2020	China	84	50.9	48	HTN, DM, IHD, Trop	Severity	★★★★★★★	[84]
Wan S	J Med Virol	21/03/2020	China	135	46.0	72	HTN, DM, IHD	Death, ARDS, I+V, AKI, Severity	★★★★★★	[85]
Zheng F	Eur Rev Med Pharm Sci	20/03/2020	China	161	45.2	80	HTN, DM, IHD	Severity	★★★★★	[86]
Shi Y	Crit Care	18/03/2020	China	487	46.0	259	HTN, DM, IHD	Death, Severity	★★★★★★	[87]
Chen T	BMJ	17/03/2020	China	274	67.0	171	HTN, DM, IHD, Trop	Death, I+V, AKI	★★★★★★★	[88]
Liu Y	medRxiv	16/03/2020	China	291	48.0	133	HTN, DM, IHD, Trop	Death, ARDS, I+V, ITU, AKI, Severity	★★★★★★	[89]
Fu L	medRxiv	16/03/2020	China	200	N/A	99	HTN, DM, IHD, Trop	Death	★★★★★★	[90]
Cao J	Clin Infect Dis	13/03/2020	China	102	52.7	53	HTN, DM, IHD, Trop	Death, ARDS, I+V, AKI	★★★★★★★	[91]
Wu C	JAMA Intern Med	13/03/2020	China	201	51.3	128	HTN, DM, IHD	Death, ARDS, I+V, ITU	★★★★★★★★★	[92]
Liu R	medRxiv	12/03/2020	China	41	39.1	17	HTN, DM, IHD, Trop	Death, ARDS, I+V, ITU, AKI,	★★★★★	[93]
Zhu F	Lancet	11/03/2020	China	191	56.3	119	HTN, DM, IHD, Trop	Death, ARDS, I+V, ITU, AKI, Severity	★★★★★★★★★	[94]
Xu H	medRxiv	08/03/2020	China	53	73.6	28	HTN, DM, IHD, Trop	Death, ARDS, I+V, ITU, AKI,	★★★★★★	[95]

Zhang G	medRxiv	06/03/2020	China	221	53.5	108	HTN, DM, IHD, Trop	Death, ARDS, I+V, AKI, Severity	★★★★★★	96
Cao M	medRxiv	06/03/2020	China	198	50.1	101	HTN, DM, IHD, Trop	Death, ITU, AKI	★★★★★★	97
Chen X	medRxiv	06/03/2020	China	291	46.3	145	HTN, DM, IHD	Death, Severity	★★★★★★	98
Bai T	Lancet Resp Med	05/03/2020	China	127	55.3	80	HTN, DM, IHD, Trop	Death, ARDS	★★★★★★★★	99
Yang H	J Peking University	04/03/2020	China	94	66.7	45	HTN, DM, IHD, Trop	Death, Severity	★★★★★★★	100
Qi D	medRxiv	03/03/2020	China	267	49.3	149	HTN, DM, IHD, Trop	Death, ARDS, I+V, ITU, Severity	★★★★★★	101
Wang Y	medRxiv	03/03/2020	China	110	N/A	48	HTN, DM	Severity	★★★★★★	102
Peng Y	Chin J Cardiovasc Dis	02/03/2020	China	112	61.3	53	HTN, DM, IHD	Death, Severity	★★★★★★	103
Wu C	medRxiv	29/02/2020	China	188	51.9	119	HTN, DM, IHD, Trop	Death, ARDS, ITU	★★★★★★★	104
Zhang JJ	Allerg	27/02/2020	China	140	56.3	71	HTN, DM, IHD	Death, Severity	★★★★★★	105
Liu Y	medRxiv	27/02/2020	China	109	54.7	59	HTN, DM, IHD	Death, ARDS	★★★★★★★	106
Liu L	medRxiv	23/02/2020	China	51	43.3	32	HTN, DM	Death, ARDS, I+V, Severity	★★★★★★★	107
Xu M	Chin Crit Care Med	23/02/2020	China	23	46.2	15	HTN, DM, IHD	Death, Severity	★★★★★	108
Wang D	JAMA	07/02/2020	China	138	55.3	75	HTN, DM, IHD, Trop	ARDS, ITU, AKI	★★★★★★	109
Huang C	Lancet	24/01/2020	China	41	49.3	30	HTN, DM, IHD, Trop	Death, ARDS, I+V, ITU, AKI,	★★★★★★	110
**Totals/Mean**				48809	56.7	27854				

* – Populations overlapped in three studies so only the most recent study, Jang et al.[24] was counted towards the total/mean numbers while Chu[41] and Hong[60] were excluded. Note: the three studies looked at different risk factors and outcomes and were not duplicated in individual meta-analyses.

Appendix C: excluded studies

#	Author	Study	Exclusion reason
1	Li[111]	Cardiovascular disease potentially contributes to the progression and poor prognosis of COVID-19	Does not relate either troponin or relevant comorbidities to outcomes of interest.
2	Zhou[112]	The clinical characteristics of myocardial injury in severe and very severe patients with 2019 novel coronavirus disease	Does not relate either troponin or relevant comorbidities to outcomes of interest.
3	Zhang[113]	Analysis of clinical characteristics and laboratory findings of 95 cases of 2019 novel coronavirus pneumonia in Wuhan, China: a retrospective analysis	Does not relate either troponin or relevant comorbidities to outcomes of interest.
4	Farahani[114]	Clinical Features of ICU Admitted and Intubated Novel Corona Virus-infected Patients in Iran	Does not relate either troponin or relevant comorbidities to outcomes of interest.
5	Klok[115]	Confirmation of the high cumulative incidence of thrombotic complications in critically ill ICU patients with COVID-19: An updated analysis	Does not relate either troponin or relevant comorbidities to outcomes of interest.
6	Li[116]	Retrospective analysis of laboratory testing in 54 patients with severe- or critical-type 2019 novel coronavirus pneumonia	Does not relate either troponin or relevant comorbidities to outcomes of interest.
7	Yang[117]	The role of essential organ-based comorbidities in the prognosis of COVID-19 infection patients	Does not relate either troponin or relevant comorbidities to outcomes of interest.
8	Liu[118]	Clinical characteristics of 30 medical workers infected with new coronavirus pneumonia	Does not relate either troponin or relevant comorbidities to outcomes of interest.
9	Medetalibeyoglu[119]	Characteristics of the initial patients hospitalized for COVID-19: a single-center report	Does not relate either troponin or relevant comorbidities to outcomes of interest.
10	Ling[120]	Clinical analysis of risk factors for severe patients with novel coronavirus pneumonia	Does not relate either troponin or relevant comorbidities to outcomes of interest.
11	Zhao[121]	Clinical characteristics of 28 patients with novel coronavirus pneumonia	Does not relate either troponin or relevant comorbidities to outcomes of interest.
12	Ma[122]	Epidemiological, Clinical, and Immunological Features of a Cluster of COVID-19–Contracted Hemodialysis Patients	Does not relate either troponin or relevant comorbidities to outcomes of interest.
13	Yaghi[123]	SARS-CoV-2 and Stroke in a New York Healthcare System	Does not relate either troponin or relevant comorbidities to outcomes of interest.
14	Fu[124]	Virologic and clinical characteristics for prognosis of severe COVID-19: a retrospective observational study in Wuhan, China	Does not relate either troponin or relevant comorbidities to outcomes of interest.
15	Colon[125]	Atrial Arrhythmias in COVID-19 Patients	Does not relate either troponin or relevant comorbidities to outcomes of interest.
16	Liu[126]	Clinical characteristics of novel coronavirus cases in tertiary hospitals in Hubei Province	Does not relate either troponin or relevant comorbidities to outcomes of interest.
17	Zhu[127]	Evaluation of organ function in patients with severe COVID-19 infections	Does not relate either troponin or relevant comorbidities to outcomes of interest.
18	Cantador[128]	Incidence and consequences of systemic arterial thrombotic events in COVID-19 patients	Does not relate either troponin or relevant comorbidities to outcomes of interest.
19	Szekely[129]	Spectrum of Cardiac Manifestations in COVID-19	Does not relate either troponin or relevant comorbidities to outcomes of interest.

20	Sud[130]	Echocardiographic Findings in Patients with COVID-19 with Significant Myocardial Injury	Does not relate either troponin or relevant comorbidities to outcomes of interest.
21	Jain[131]	Indications for and Findings on Transthoracic Echocardiography in COVID-19	Does not relate either troponin or relevant comorbidities to outcomes of interest.
22	Li[132]	Sex differences in clinical findings among patients with coronavirus disease 2019 (COVID-19) and severe condition	Does not relate either troponin or relevant comorbidities to outcomes of interest.
23	Chen[133]	Analysis of myocardial injury in patients with COVID-19 and association between concomitant cardiovascular diseases and severity of COVID-19	Includes patients under 18 years old.
24	Sun[134]	Clinical analysis of 150 cases of 2019 novel coronavirus infection in Nanyang City, Henan Province	Includes patients under 18 years old.
25	Sabatino[135]	COVID-19 and Congenital Heart Disease: Results from a Nationwide Survey	Includes patients under 18 years old.
26	López-Otero[136]	Impact of angiotensin-converting enzyme inhibitors and angiotensin receptor blockers on COVID-19 in a western population. CARDIOVID registry	Includes patients under 18 years old.
27	Escalera-Antezana[137]	Risk factors for mortality in patients with Coronavirus Disease 2019 (COVID-19) in Bolivia: An analysis of the first 107 confirmed cases	Includes patients under 18 years old.
28	Gao[138]	The epidemiological characteristics of 2019 novel coronavirus diseases (COVID-19) in Jingmen, Hubei, China	Includes patients under 18 years old.
29	Kim[139]	The Correlation of Comorbidities on the Mortality in Patients with COVID-19: an Observational Study Based on the Korean National Health Insurance Big Data	Includes patients under 18 years old.
30	Luo[140]	Prealbumin as a Predictor of Prognosis in Patients With Coronavirus Disease 2019	Includes patients under 18 years old.
31	Garcia[141]	Prognostic factors associated with mortality risk and disease progression in 639 critically ill patients with COVID-19 in Europe: Initial report of the international RISC-19-ICU prospective observational cohort	Includes patients under 18 years old.
32	Ruan[142]	Clinical predictors of mortality due to COVID-19 based on an analysis of data of 150 patients from Wuhan, China	Includes patients under 18 years old.
33	Liu[143]	Neutrophil-to-Lymphocyte Ratio Predicts Severe Illness Patients with 2019 Novel Coronavirus in the Early Stage	Includes patients under 18 years old.
34	Hui[144]	Clinical and radiographic features of cardiac injury in patients with 2019 novel coronavirus pneumonia	Includes patients under 18 years old.
35	Palmieri[145]	Clinical Characteristics of Hospitalized Individuals Dying With COVID-19 by Age Group in Italy	Includes patients under 18 years old.
36	Bhatraju[146]	Covid-19 in Critically Ill Patients in the Seattle Region — Case Series	Selects for ICU patients.
37	Fan[147]	Cardiac injuries in patients with coronavirus disease 2019: Not to be ignored	Selects for ICU patients.

38	Zheng[148]	Clinical characteristics of 34 COVID-19 patients admitted to intensive care unit in Hangzhou, China	Selects for ICU patients.
39	Yang[149]	Clinical course and outcomes of critically ill patients with SARS-CoV-2 pneumonia in Wuhan, China: a single-centered, retrospective, observational study	Selects for ICU patients.
40	Yu[150]	Patients with COVID-19 in 19 ICUs in Wuhan, China: a cross-sectional study	Selects for ICU patients.
41	Ge[151]	Cardiac Structural and Functional Characteristics in Patients with Coronavirus Disease 2019: A Serial Echocardiographic Study	Selects for ICU patients.
42	Xu[152]	Clinical findings in critical ill patients infected with SARS-Cov-2 in Guangdong Province, China: a multi-center, retrospective, observational study	Selects for ICU patients.
43	Zou[153]	Acute Physiology and Chronic Health Evaluation II Score as a Predictor of Hospital Mortality in Patients of Coronavirus Disease 2019	Selects for ICU patients.
44	Xu[154]	Clinical course and predictors of 60-day mortality in 239 critically ill patients with COVID-19: a multicenter retrospective study from Wuhan, China	Selects for ICU patients.
45	Shang[155]	Scoring systems for predicting mortality for severe patients with COVID-19	Selects for ICU patients.
46	Ferrando[156]	Patient characteristics, clinical course and factors associated to ICU mortality in critically ill patients infected with SARS-CoV-2 in Spain: A prospective, cohort, multicentre study	Selects for ICU patients.
47	Hu[157]	Clinical features of critically ill patients with COVID-19 infection in China	Selects for ICU patients.
48	Zhou[158]	Potential benefits of precise corticosteroids therapy for severe 2019-nCoV pneumonia	Selects for ICU patients.
49	Shi[159]	Characteristics and clinical significance of myocardial injury in patients with severe coronavirus disease 2019	Selects for severe or critical patients.
50	He[160]	Impact of complicated myocardial injury on the clinical outcome of severe or critically ill COVID-19 patients	Selects for severe or critical patients.
51	Li[161]	Clinical Characteristics and Outcomes of 74 Patients With Severe or Critical COVID-19	Selects for severe or critical patients.
52	Yan[162]	Clinical characteristics and outcomes of patients with severe covid-19 with diabetes	Selects for severe or critical patients.
53	Huang[163]	Clinical Characteristics and Predictors of Disease Progression in Severe Patients with COVID-19 Infection in Jiangsu Province, China: A Descriptive Study	Selects for severe or critical patients.
54	Cummings[164]	Epidemiology, clinical course, and outcomes of critically ill adults with COVID-19 in New York City: a prospective cohort study	Selects for severe or critical patients.
55	Chen[165]	Cardiovascular manifestations in severe and critical patients with COVID-19	Selects for severe or critical patients.

56	Xu[166(p2)]	The impact of type 2 diabetes and its management on the prognosis of patients with severe COVID-19	Selects for severe or critical patients.
57	Pan[167]	Factors associated with death outcome in patients with severe coronavirus disease-19 (COVID-19): a case-control study	Selects for severe or critical patients.
58	Chen[168]	The characteristics and outcomes of 681 severe cases with COVID-19 in China	Selects for severe or critical patients.
59	Arentz[169]	Characteristics and Outcomes of 21 Critically Ill Patients With COVID-19 in Washington State	Selects for severe or critical patients.
60	Wang[170]	Clinical Course and Outcomes of 344 Intensive Care Patients with COVID-19	Selects for severe or critical patients.
61	Yang[171(p92)]	Analysis of 92 deceased patients with COVID-19	Selects for deceased patients.
62	Du[172]	Clinical Features of 85 Fatal Cases of COVID-19 from Wuhan. A Retrospective Observational Study	Selects for deceased patients.
63	Shi[173]	Clinical characteristics of 101 COVID-19 nonsurvivors in Wuhan, China: a retrospective study	Selects for deceased patients.
64	Zhang[174]	Clinical characteristics of 82 death cases with COVID-19	Selects for deceased patients.
65	Li[175]	Clinical characteristics of 25 death cases with COVID-19: A retrospective review of medical records in a single medical center, Wuhan, China	Selects for deceased patients.
66	Huang[176]	Clinical characteristics of 36 non-survivors with COVID-19 in Wuhan, China	Selects for deceased patients.
67	Du[177]	Hospitalization and Critical Care of 109 Decedents with COVID-19 Pneumonia in Wuhan, China	Selects for deceased patients.
68	Gao[178]	Prognostic value of NT-proBNP in patients with severe COVID-19	Reports data only as hazard ratio or odds ratio with no reliable method to convert to raw data.
69	Palaiodimos[179]	Severe obesity, increasing age and male sex are independently associated with worse in-hospital outcomes, and higher in-hospital mortality, in a cohort of patients with COVID-19 in the Bronx, New York	Reports data only as hazard ratio or odds ratio with no reliable method to convert to raw data.
70	Zhao[180]	Comparison of clinical characteristics and outcomes of patients with coronavirus disease 2019 at different ages	Reports data only as hazard ratio or odds ratio with no reliable method to convert to raw data.
71	Li[181]	Clinical features and short-term outcomes of elderly patients with COVID-19	Selects for patients over age of 60.
72	Wang[182]	Coronavirus disease 2019 in elderly patients: Characteristics and prognostic factors based on 4-week follow-up	Selects for patients over age of 60.
73	Li[183]	Clinical characteristics of 312 hospitalized older patients with COVID-19 in Wuhan, China	Selects for patients over age of 65.
74	Wang[184]	Clinical characteristics of 28 patients with diabetes and covid-19 in Wuhan, China	Selects for diabetic patients.
75	Li[185]	Baseline characteristics and risk factors for short-term outcomes in 132 COVID-19 patients	Selects for diabetic patients.

		with diabetes in Wuhan China: A retrospective study	
76	Louhaichi[186]	Features of patients with 2019 novel coronavirus admitted in a pneumology department: The first retrospective Tunisian case series	Does not relate either troponin or relevant comorbidities to outcomes of interest.
77	Aggarwal[187]	Clinical features, laboratory characteristics, and outcomes of patients hospitalized with coronavirus disease 2019 (COVID-19): Early report from the United States	Does not relate either troponin or relevant comorbidities to outcomes of interest.
78	Su[188]	Two consecutive myocardial tissue insults for inpatients with COVID-19	Data was incomplete and could not be used for meta-analysis.
79	Yang[189]	Clinical characteristics and outcomes of cancer patients with COVID-19	Selects for cancer patients.
80	Xie[190]	Clinical characteristics of non-ICU hospitalized patients with coronavirus disease 2019 and liver injury: A retrospective study	Selects for non-ICU and alive patients.
81	Si[191]	Death, discharge and arrhythmias among patients with COVID-19 and cardiac injury	Selects for patients with elevated troponin.
82	Pierce-Williams[192]	Clinical course of severe and critical coronavirus disease 2019 in hospitalized pregnancies: a United States cohort study	Selects for pregnant patients.

Appendix D: Risk of Bias (Newcastle-Ottawa Score)

#	Author	1. Representativeness of exposed cohort -	2. Selection of non-exposed cohort - drawn from same cohort as exposed	3. Ascertainment of exposure	4. Demonstration that outcome of interest was not present at start of study -	1. Adjusts for age and sex	2. Adjusts for any of: HTN, DM, IHD, chronic resp, CKD, malignancy	1. Assessment of outcome	2. Length of follow-up - 1 star if f/u until discharge	3. Adequacy of follow-up	Total
1	Franks C	★	★	★	★			★	★	★	★★★★★★★
2	Deng Q	★	★	★	★			★			★★★★★
3	Chen X	★	★	★	★			★	★	★	★★★★★★★
4	Shang J	★	★	★	★	★	★	★		★	★★★★★★★★
5	Aloisio E	★	★	★	★	★		★	★	★	★★★★★★★★
6	Ferrante F	★	★	★	★	★		★		★	★★★★★★★
7	Yang X	★	★	★	★	★	★	★		★	★★★★★★★★
8	Ni W	★	★		★	★	★	★	★	★	★★★★★★★★
9	Liu D	★	★	★	★			★		★	★★★★★★
10	van den Heuvel F	★	★	★	★			★		★	★★★★★★
11	Pelayo J	★	★	★	★	★	★	★		★	★★★★★★★★
12	Lodigiani C	★	★	★	★			★		★	★★★★★★
13	Sun L	★	★	★	★	★	★	★	★	★	★★★★★★★★★
14	McCullough S	★	★	★	★	★		★		★	★★★★★★★
15	Zhao Y	★	★	★	★			★		★	★★★★★★
16	Bonetti G	★	★	★	★			★	★	★	★★★★★★★
17	Aggarwal AS	★	★	★	★	★	★	★		★	★★★★★★★★

18	Iaccarino D	★	★	★	★	★	★	★		★	★★★★★★★★
19	Barman HA	★	★	★	★	★	★	★		★	★★★★★★★★
20	Lorente RA	★	★	★	★	★	★	★	★	★	★★★★★★★★★
21	Cao Z	★	★	★	★	★	★	★		★	★★★★★★★★
22	Suleyman F	★	★	★	★	★	★	★	★	★	★★★★★★★★★
23	Mani V	★	★	★	★	★	★	★		★	★★★★★★★★
24	Jang J	★	★	★	★	★	★	★		★	★★★★★★★★
25	Nie S		★	★	★	★	★	★	★	★	★★★★★★★★
26	Xie Y	★	★	★	★	★	★	★		★	★★★★★★★★
27	Ciceri F	★	★	★	★	★	★	★		★	★★★★★★★★
28	Gopal P	★	★	★	★			★		★	★★★★★★
29	Mikami T	★	★	★	★	★	★	★	★	★	★★★★★★★★★
30	Okoh S		★	★	★	★	★	★	★	★	★★★★★★★★
31	Violi F	★	★	★	★			★			★★★★★
32	Deng Y	★	★	★	★			★	★	★	★★★★★★★
33	Feng Y	★	★	★	★			★		★	★★★★★★
34	Yang AP	★	★	★	★			★		★	★★★★★★
35	Zengin A	★	★	★	★			★		★	★★★★★★
36	Wang CZ	★	★	★	★			★		★	★★★★★★
37	Rath D	★	★	★	★		★	★		★	★★★★★★★
38	Garibaldi B	★	★	★	★	★	★	★		★	★★★★★★★★
39	Yang Q	★	★	★	★			★		★	★★★★★★
40	Petrilli C	★	★	★	★	★	★	★	★	★	★★★★★★★★★
41	Chu S	★	★	★	★	★	★	★		★	★★★★★★★★

42	Zhang Y	★	★	★	★			★		★	★★★★★★
43	Schiller M	★	★	★	★			★		★	★★★★★★
44	Lu Y	★	★	★	★			★	★	★	★★★★★★★
45	Hirsch J	★	★	★	★	★	★	★		★	★★★★★★★★
46	Kuno T	★	★	★	★	★		★		★	★★★★★★★
47	Zhang J	★	★	★	★			★		★	★★★★★★
48	Inciardi R	★	★	★	★			★	★	★	★★★★★★★
49	Borobia A	★	★	★	★			★		★	★★★★★★
50	DU RH	★	★	★	★		★	★	★	★	★★★★★★★★
51	Hu L	★	★	★	★	★	★	★		★	★★★★★★★★
52	Wang K	★		★	★	★	★	★	★	★	★★★★★★★★
53	Guan WJ	★	★	★	★			★		★	★★★★★★
54	Wei JF	★	★	★	★		★	★		★	★★★★★★★
55	Wang D	★	★	★	★	★	★	★	★	★	★★★★★★★★★
56	Feng X	★	★	★	★	★	★	★		★	★★★★★★★★
57	Zhao X	★	★	★	★			★		★	★★★★★★
58	Zhang J	★	★	★	★			★		★	★★★★★★
59	Tomlins J	★	★	★	★			★		★	★★★★★★
60	Hong KS	★	★	★	★			★		★	★★★★★★
61	Zhu Z	★	★	★	★	★	★	★		★	★★★★★★★★
62	Liu J	★	★	★	★			★		★	★★★★★★
63	Chen X	★	★	★	★			★	★	★	★★★★★★★
64	Wang L	★	★	★	★	★		★	★	★	★★★★★★★★
65	Li X	★	★	★	★	★	★	★		★	★★★★★★★★

66	He R	★	★	★	★			★		★	★★★★★★
67	Fang X	★	★	★	★			★		★	★★★★★★
68	Mao L	★	★	★	★			★		★	★★★★★★
69	Gu T	★		★	★	★	★	★	★	★	★★★★★★★★
70	Dreher M	★	★	★	★			★		★	★★★★★★
71	Wang L	★	★	★	★			★		★	★★★★★★
72	Han H	★	★	★	★			★		★	★★★★★★
73	Fan L	★	★	★	★			★	★	★	★★★★★★★
74	Li YK	★	★	★	★			★		★	★★★★★★
75	Chen G	★	★	★	★			★	★	★	★★★★★★★
76	Guo T	★	★	★	★		★	★	★	★	★★★★★★★★
77	Li K	★	★	★	★	★	★	★	★	★	★★★★★★★★★
78	Zhou Y	★	★	★	★	★		★		★	★★★★★★★
79	Han Y	★	★	★	★	★		★		★	★★★★★★★
80	Shi S	★	★	★	★	★	★	★		★	★★★★★★★★
81	Zhang	★	★	★	★		★	★		★	★★★★★★★
82	Luo X	★	★	★	★			★	★	★	★★★★★★★
83	Yan S	★	★	★	★			★		★	★★★★★★
84	Ma KL	★	★	★	★	★		★		★	★★★★★★★
85	Wan S	★	★	★	★			★		★	★★★★★★
86	Zheng F	★	★		★			★		★	★★★★★
87	Shi Y	★	★	★	★			★		★	★★★★★★
88	Chen T	★	★	★	★			★	★	★	★★★★★★★
89	Liu Y	★	★	★	★			★		★	★★★★★★

90	Fu L	★	★	★	★			★	★	★	★★★★★★
91	Cao J	★	★	★	★			★	★	★	★★★★★★★
92	Wu C	★	★	★	★	★	★	★	★	★	★★★★★★★★★
93	Liu R		★	★	★			★		★	★★★★★
94	Zhu F	★	★	★	★	★	★	★	★	★	★★★★★★★★★
95	Xu H	★	★	★	★			★		★	★★★★★★
96	Zhang G	★	★	★	★			★		★	★★★★★★
97	Cao M	★	★	★	★			★		★	★★★★★★
98	Chen X	★	★	★	★			★		★	★★★★★★
99	Bai T	★	★	★	★	★		★	★	★	★★★★★★★★
100	Yang H	★	★	★	★			★	★	★	★★★★★★★
101	Qi D	★	★	★	★			★		★	★★★★★★
102	Wang Y	★	★	★	★			★		★	★★★★★★
103	Peng Y	★	★		★			★	★	★	★★★★★★
104	Wu C	★	★	★	★			★	★	★	★★★★★★★
105	Zhang JJ	★	★	★	★			★		★	★★★★★★
106	Liu Y	★	★	★	★			★	★	★	★★★★★★★
107	Liu L	★	★	★	★			★	★	★	★★★★★★★
108	Xu M	★	★		★			★		★	★★★★★
109	Wang D	★	★	★	★			★		★	★★★★★★
110	Huang C	★	★	★	★			★		★	★★★★★★

Appendix D.1

Criteria for grading the Newcastle-Ottawa Score for each study.
**Selection**
**1) Representativeness of the exposed cohort**
a) truly representative of the average adult COVID-19 patient in the community ★
b) somewhat representative of the average adult COVID-19 patient in the community ★
c) selected group of users eg nurses, volunteers, dialysis patients, pregnant patients, ITU only etc.
d) no description of the derivation of the cohort
**2) Selection of the non exposed cohort**
a) drawn from the same community as the exposed cohort ★
b) drawn from a different source
c) no description of the derivation of the non exposed cohort
**3) Ascertainment of exposure**
a) secure record (eg surgical records) ★
b) structured interview ★
c) written self report
d) no description
**4) Demonstration that outcome of interest was not present at start of study**
a) yes (i.e. no prior history of COVID-19 - although not demonstrated, reasonable assumption given novel disease) ★
b) no
**Comparability**
**1) Comparability of cohorts on the basis of the design or analysis**
a) study controls for age and sex (select the most important factor) ★
b) study controls for any additional factor: HTN, DM, IHD, chronic respiratory disease, chronic kidney disease, active malignancy (e.g. has run a multivariate analysis or other analysis that adjusts for this) ★
**Outcome**
**1) Assessment of outcome**
a) independent blind assessment ★
b) record linkage ★ (e.g. electronic health records)

c) self report
d) no description
**2) <u>Was follow-up long enough for outcomes to occur</u>**
a) yes (follow-up until discharge) ★
b) no
**3) <u>Adequacy of follow up of cohorts</u>**
a) complete follow up - all subjects accounted for ★
b) subjects lost to follow up unlikely to introduce bias -small number lost- > 20% follow up, or description provided of those lost ★
c) follow up rate < 20% and no description of those lost
d) no statement

Appendix E: GRADE assessment

Outcome	Risk factor	n of studies	n of patients (total)	Initial Study Grade	Risk of bias	Imprecision	Inconsistency	Indirectness	Publication Bias	Total	Justification
Death	DM	40	18979	2	-1	-1	0	0	0	0	Downgraded for risk of bias (inadequate follow up in large scale studies) and imprecision (lower boundary of many studies crossing line of no effect)
	HTN	37	17995	2	-1	1	0	0	0	2	Risk of bias downgrade (-1) as studies with large sample size had inadequate follow-up period. Approximately 50% of studies did not account for confounders, however studies with large sample size adjusted for confounder effect, therefore, not downgraded for this element. Upgraded for large effect size
	IHD	37	19968	2	-1	1	0	0	0	2	Downgraded for risk of bias (inadequate follow up in large scale studies). Upgraded for large effect size
	Trop	35	21707	2	-1	1	0	0	0	2	Downgraded for risk of bias (inadequate follow-up in large scale studies). Upgraded for large effect size.
ARDS	DM	7	1428	2	0	0	0	0	0	2	
	HTN	4	476	2	0	-1	0	0	0	1	Downgraded (-1) for imprecision due to wide confidence intervals
	IHD	2	310	2	0	-1	-1	0	0	0	Only 2 studies in this analysis. Downgraded (-1) for imprecision due to small study size and wide confidence intervals. Downgraded (-1) for inconsistency due to variable effect size
	Trop	9	2189	2	0	0	1	0	0	3	Upgrade for magnitude of effect

Outcome	Risk factor	n of studies	n of patients (total)	Initial Study Grade	Risk of bias	Imprecision	Inconsistency	Indirectness	Publication Bias	Total	Justification
Mechanical Ventilation	DM	4	1345	2	0	0	0	0	0	2	
	HTN	1	393	x	x	x	x	x	x	x	GRADE assessment not performed - only 1 study in meta-analysis
	IHD	2	8831	2	0	0	0	0	0	2	
	Trop	12	10424	2	0	1	0	0	0	3	Upgrade for magnitude of effect
ITU	DM	11	2487	2	0	-1	-1	0	0	0	Downgraded (-1) for imprecision due to wide confidence intervals. Downgraded (-1) for inconsistency due to variable effect size
	HTN	10	1891	2	0	-1	-1	0	0	0	Downgraded (-1) for imprecision due to small study size and wide confidence intervals. Downgraded for inconsistency (-1) due to variation in point estimates and limited overlapping of confidence intervals
	IHD	10	1891	2	0	-1	-1	0	0	0	Downgraded (-1) for imprecision due to small study size and wide confidence intervals. Downgraded for inconsistency (-1) due to variation in point estimates and limited overlapping of confidence intervals
	Trop	14	2753	2	-1	0	1	0	0	2	Downgraded (-1) for risk of bias due to short length of follow-up. Upgraded for large magnitude of effect

Outcome	Risk factor	n of studies	n of patients (total)	Initial Study Grade	Risk of bias	Imprecision	Inconsistency	Indirectness	Publication Bias	Total	Justification
AKI	DM	6	7018	2	0	-1	0	0	0	1	Downgraded (-1) for imprecision due to wide confidence intervals
	HTN	3	6066	2	0	-1	-1	0	0	0	Downgraded (-1) for imprecision due to small study size and wide confidence intervals. Downgraded (-1) for inconsistency due to no overlap of confidence intervals
	IHD	3	6066	2	0	0	0	0	0	2	
	Trop	7	1777	2	0	0	1	0	0	3	Upgrade for magnitude of effect
Severe disease	DM	43	11495	2	0	-1	0	0	0	1	Downgraded (-1) on imprecision due to small study size and wide confidence intervals
	HTN	41	10653	2	-1	-1	0	0	0	0	Downgraded (-1) on risk of bias for length of follow-up. Downgraded (-1) for imprecision due to small study size and wide confidence intervals
	IHD	33	10149	2	0	-1	0	0	0	1	Downgraded (-1) on imprecision due to wide confidence intervals
	Trop	18	4731	2	-1	0	1	0	0	2	Downgraded (-1) on risk of bias due to length of follow-up. Upgrade for magnitude of effect

Appendix F: Publication bias

Diabetes Mellitus

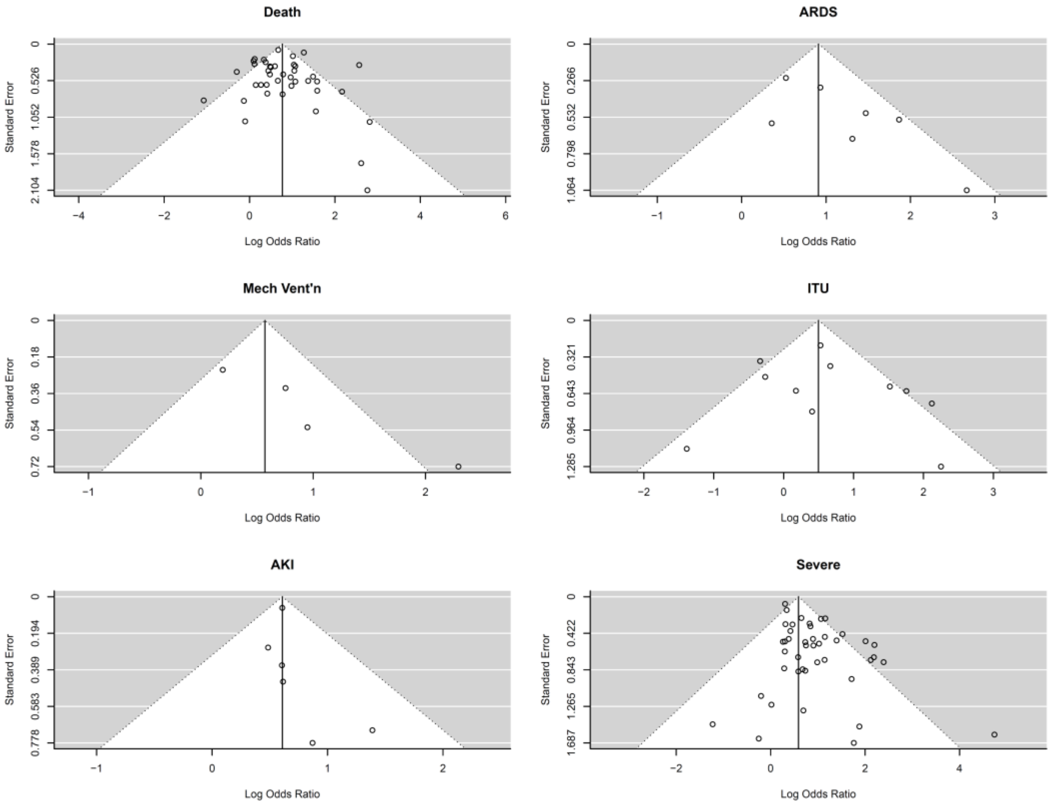

Hypertension

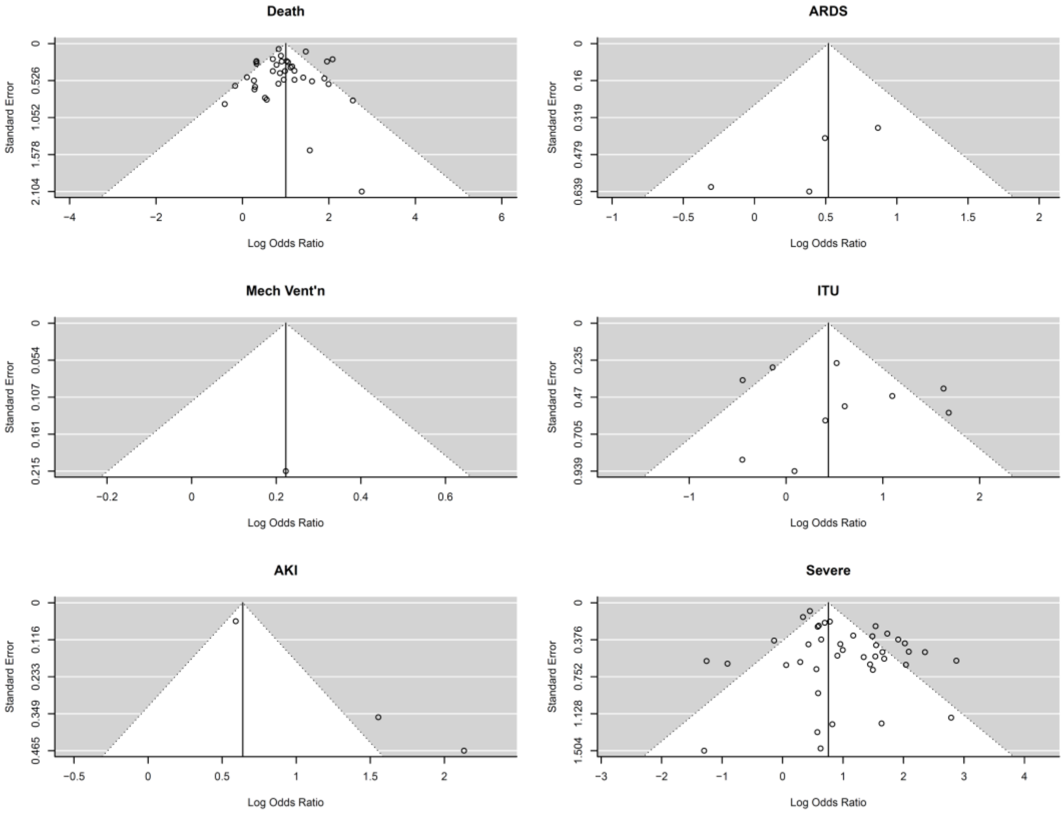

Appendix F: Publication bias

Ischaemic Heart Disease

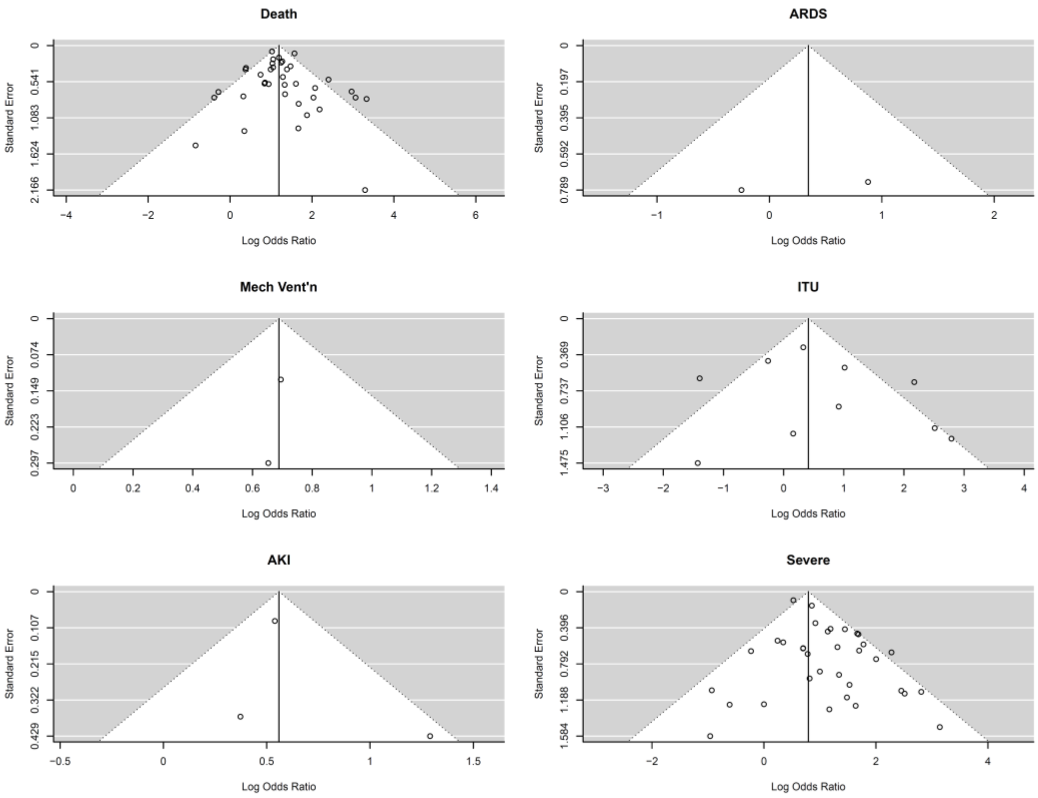

Myocardial Injury

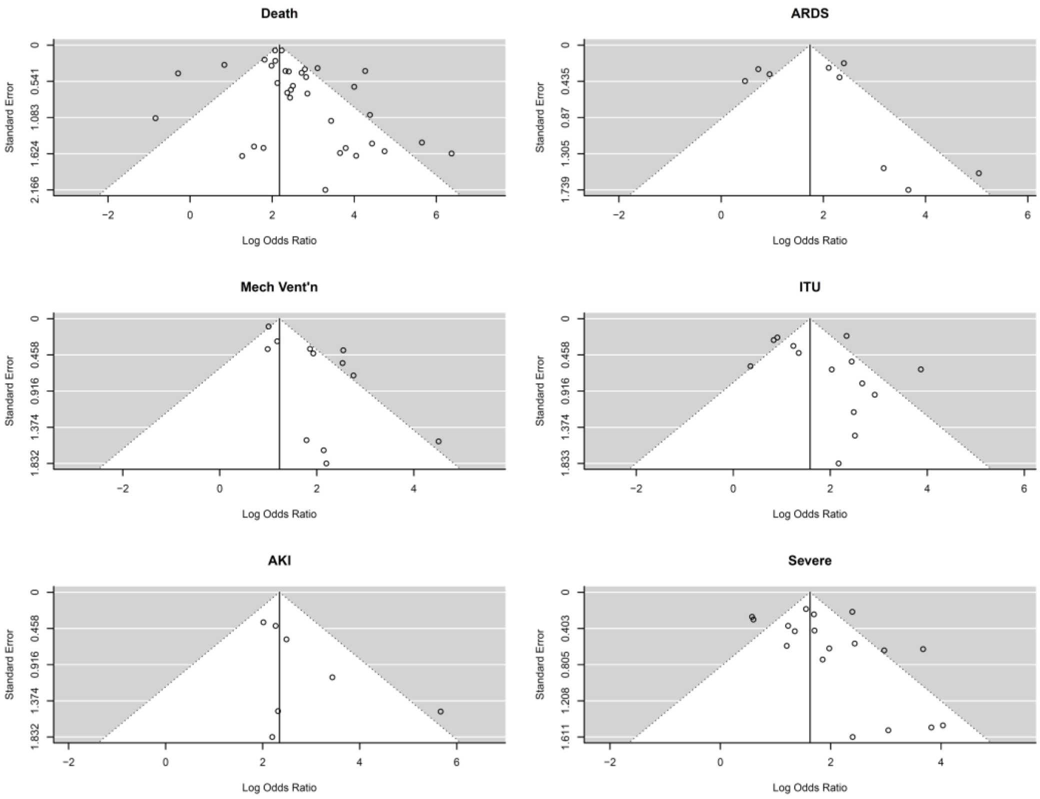

Appendix G: MOOSE Checklist for Meta-analyses of Observational Studies

Item No	Recommendation	Reported on Page No
Reporting of background should include		
1	Problem definition	5
2	Hypothesis statement	5
3	Description of study outcome(s)	6 - 7
4	Type of exposure or intervention used	6 - 7
5	Type of study designs used	-
6	Study population	6
Reporting of search strategy should include		
7	Qualifications of searchers (eg, librarians and investigators)	Supp 1
8	Search strategy, including time period included in the synthesis and key words	Supp 1-4
9	Effort to include all available studies, including contact with authors	Supp 1-4
10	Databases and registries searched	Supp 1-4
11	Search software used, name and version, including special features used (eg, explosion)	Supp 1-4
12	Use of hand searching (eg, reference lists of obtained articles)	Supp 5-10
13	List of citations located and those excluded, including justification	Supp 5-15
14	Method of addressing articles published in languages other than English	6
15	Method of handling abstracts and unpublished studies	6
16	Description of any contact with authors	-
Reporting of methods should include		
17	Description of relevance or appropriateness of studies assembled for assessing the hypothesis to be tested	6 - 7
18	Rationale for the selection and coding of data (eg, sound clinical principles or convenience)	6 - 7
19	Documentation of how data were classified and coded (eg, multiple raters, blinding and interrater reliability)	6 - 7
20	Assessment of confounding (eg, comparability of cases and controls in studies where appropriate)	-
21	Assessment of study quality, including blinding of quality assessors, stratification or regression on possible predictors of study results	6 – 7, Supp 16 - 20, 23-25
22	Assessment of heterogeneity	7
23	Description of statistical methods (eg, complete description of fixed or random effects models, justification of whether the chosen models account for predictors of study results, dose-response models, or cumulative meta-analysis) in sufficient detail to be replicated	7
24	Provision of appropriate tables and graphics	
Reporting of results should include		

25	Graphic summarizing individual study estimates and overall estimate	8
26	Table giving descriptive information for each study included	Supp 5 - 10
27	Results of sensitivity testing (eg, subgroup analysis)	-
28	Indication of statistical uncertainty of findings	8 – 9

Item No	Recommendation	Reported on Page No
Reporting of discussion should include		
29	Quantitative assessment of bias (eg, publication bias)	Supp 26 - 27
30	Justification for exclusion (eg, exclusion of non-English language citations)	Supp 11 - 15
31	Assessment of quality of included studies	Supp 16 - 22
Reporting of conclusions should include		
32	Consideration of alternative explanations for observed results	10 - 11
33	Generalization of the conclusions (ie, appropriate for the data presented and within the domain of the literature review)	10 - 11
34	Guidelines for future research	13 - 14
35	Disclosure of funding source	

From: Stroup DF, Berlin JA, Morton SC, et al, for the Meta-analysis Of Observational Studies in Epidemiology (MOOSE) Group. Meta-analysis of Observational Studies in Epidemiology. A Proposal for Reporting. *JAMA*. 2000;283(15):2008-2012. doi: 10.1001/jama.283.15.2008.

Appendix H: References (Supplemental)

1. Franks CE, Scott MG, Farnsworth CW. Elevated Cardiac Troponin I Is Associated with Poor Outcomes in COVID-19 Patients at an Academic Medical Center in Midwestern USA. *The journal of applied laboratory medicine*. Published online 2020. https://academic.oup.com/jalm/advance-article/doi/10.1093/jalm/jfaa092/5850405

2. Deng Q, Hu B, Zhang Y, et al. Suspected myocardial injury in patients with COVID-19: Evidence from front-line clinical observation in Wuhan, China. *International journal of cardiology*. Published online 2020. https://auth.elsevier.com/ShibAuth/institutionLogin?entityID=https://idp.eng.nhs.uk/openathens&appReturnURL=https%3A%2F%2Fwww.clinicalkey.com%2Fcontent%2FplayBy%2Fdoi%2F%3Fv%3D10.1016%2Fj.ijcard.2020.03.087

3. Chen X, Yan L, Fei Y, Zhang C. Laboratory abnormalities and risk factors associated with in-hospital death in patients with severe COVID-19. *Journal of clinical laboratory analysis*. Published online 2020:e23467-e23467.

4. Shang J, Wang Q, Zhang H, et al. The Relationship between Diabetes Mellitus and COVID-19 Prognosis: A Retrospective Cohort Study in Wuhan, China. *The American journal of medicine*. Published online 2020. https://auth.elsevier.com/ShibAuth/institutionLogin?entityID=https://idp.eng.nhs.uk/openathens&appReturnURL=https%3A%2F%2Fwww.clinicalkey.com%2Fcontent%2FplayBy%2Fdoi%2F%3Fv%3D10.1016%2Fj.amjmed.2020.05.033

5. Aloisio E, Chibireva M, Serafini L, et al. A comprehensive appraisal of laboratory biochemistry tests as major predictors of COVID-19 severity. *Archives of pathology & laboratory medicine*. Published online 2020.

6. Ferrante G, Fazzari F, Cozzi O, et al. Risk factors for myocardial injury and death in patients with COVID-19: insights from a cohort study with chest computed tomography. *Cardiovascular research*. Published online 2020.

7. Xie Y, Chen S, Wang X, et al. Early Diagnosis and Clinical Significance of Acute Cardiac Injury - Under the Iceberg: A Retrospective Cohort Study of 619 Non-critically Ill Hospitalized COVID-19 Pneumonia Patients. *medRxiv*. Published online July 7, 2020:2020.07.06.20147256. doi:10.1101/2020.07.06.20147256

8. Ni W, Yang X, Liu J, et al. Acute Myocardial Injury at Hospital Admission is Associated with All-cause Mortality in COVID-19. *Journal of the American College of Cardiology*. Published online 2020. https://auth.elsevier.com/ShibAuth/institutionLogin?entityID=https://idp.eng.nhs.uk/openathens&appReturnURL=https%3A%2F%2Fwww.clinicalkey.com%2Fcontent%2FplayBy%2Fdoi%2F%3Fv%3D10.1016%2Fj.jacc.2020.05.007

9. Liu D, Yang Q, Chen W, et al. Troponin I, a risk factor indicating more severe pneumonia among patients with novel coronavirus infected pneumonia. *Clinical Infection in Practice*. 2020;7-8:100037. doi:10.1016/j.clinpr.2020.100037

10. van den Heuvel FMA, Vos JL, Koop Y, et al. Cardiac function in relation to myocardial injury in hospitalised patients with COVID-19. *Netherlands heart journal : monthly journal of the Netherlands Society of Cardiology and the Netherlands Heart Foundation*. Published online 2020. http://europepmc.org/search?query=(DOI:10.1007/s12471-020-01458-2)

11. Pelayo J, Lo KB, Bhargav R, et al. Clinical Characteristics and Outcomes of Community- and Hospital-Acquired Acute Kidney Injury with COVID-19 in a US Inner City Hospital System. *Cardiorenal medicine*. 2020;10(4):223-231.

12. Lodigiani C, Iapichino G, Carenzo L, et al. Venous and arterial thromboembolic complications in COVID-19 patients admitted to an academic hospital in Milan, Italy. *Thrombosis research*. 2020;191:9-14.

13. Sun L, Song F, Shi N, et al. Combination of four clinical indicators predicts the severe/critical symptom of patients infected COVID-19. *Journal of clinical virology : the official publication of the Pan American Society for Clinical Virology*. 2020;128:104431-104431.

14. McCullough SA, Goyal P, Krishnan U, Choi JJ, Safford MM, Okin PM. Electrocardiographic Findings in Coronavirus Disease-19: Insights on Mortality and Underlying Myocardial Processes. *Journal of cardiac failure*. 2020 Jul; 26(7):626-632.

15. Zhao Y, Zhou J, Pan L, et al. Detection and analysis of clinical features of patients with different COVID-19 types. *Journal of medical virology*. Published online 2020. https://go.openathens.net/redirector/nhs?url=https%3A%2F%2Fdoi.org%2F10.1002%2Fjmv.26225

16. Bonetti G, Manelli F, Patroni A, et al. Laboratory predictors of death from coronavirus disease 2019 (COVID-19) in the area of Valcamonica, Italy. *Clinical chemistry and laboratory medicine*. 2020;58(7):1100-1105.

17. Aggarwal A, Shrivastava A, Kumar A, Ali A. Clinical and Epidemiological Features of SARS-CoV-2 Patients in SARI Ward of a Tertiary Care Centre in New Delhi. *The Journal of the Association of Physicians of India*. 2020;68(7):19-26.

18. Iaccarino G, Grassi G, Borghi C, et al. Age and Multimorbidity Predict Death Among COVID-19 Patients: Results of the SARS-RAS Study of the Italian Society of Hypertension. *Hypertension (Dallas, Tex : 1979)*. 2020;76(2):366-372.

19. Barman HA, Atici A, Sahin I, et al. Prognostic significance of cardiac injury in COVID-19 patients with and without coronary artery disease. *Coronary artery disease*. Published online 2020. doi: 10.1097/MCA.0000000000000914

20. Lorente-Ros A, Monteagudo Ruiz JM, Rincón LM, et al. Myocardial injury determination improves risk stratification and predicts mortality in COVID-19 patients. *Cardiology journal*. Published online 2020. https://journals.viamedica.pl/cardiology_journal/article/download/CJ.a2020.0089/51216

21. Cao Z, Li T, Liang L, et al. Clinical characteristics of Coronavirus Disease 2019 patients in Beijing, China. *PloS one*. 2020;15(6):e0234764-e0234764.

22. Suleyman, Fadel RA, Malette KM, et al. Clinical Characteristics and Morbidity Associated With Coronavirus Disease 2019 in a Series of Patients in Metropolitan Detroit. *JAMA Network Open*. 2020;3(6). https://jamanetwork.com/journals/jamanetworkopen/articlepdf/2767216/suleyman_2020_oi_200467.pdf

23. Mani VR, Kalabin A, Valdivieso SC, Murray-Ramcharan M, Donaldson B. At the epicenter of the American Coronavirus outbreak - New York inner city hospital COVID-19 experience and current data: a retrospective analysis. *Journal of medical Internet research*. Published online 2020. http://europepmc.org/search?query=(DOI:10.2196/20548)

24. Jang JG, Hur J, Choi EY, Hong KS, Lee W, Ahn JH. Prognostic Factors for Severe Coronavirus Disease 2019 in Daegu, Korea. *Journal of Korean medical science*. 2020;35(23):e209-e209.

25. Nie S-F, Yu M, Xie T, et al. Cardiac Troponin I is an Independent Predictor for Mortality in Hospitalized Patients with Coronavirus Disease 2019. *Circulation*. Published online 2020. http://ovidsp.ovid.com/ovidweb.cgi?T=JS&PAGE=fulltext&D=ovft&CSC=Y&NEWS=N&SEARCH=%2210.1161/CIRCULATIONAHA.120.048789%22.di

26. Xie Y, You Q, Wu C, et al. Impact of Cardiovascular Disease on Clinical Characteristics and Outcomes of Coronavirus Disease 2019 (COVID-19). *Circulation journal : official journal of the Japanese Circulation Society*. Published online 2020. https://www.jstage.jst.go.jp/article/circj/advpub/0/advpub_CJ-20-0348/_pdf

27. Fabio C, Antonella C, Patrizia R-Q, et al. Early predictors of clinical outcomes of COVID-19 outbreak in Milan, Italy. *Clinical Immunology*. 2020;217:108509. doi:10.1016/j.clim.2020.108509

28. Goyal P, Choi JJ, Pinheiro LC, et al. Clinical Characteristics of Covid-19 in New York City. *New England Journal of Medicine*. 2020;382(24):2372-2374. doi:10.1056/NEJMc2010419

29. Mikami T, Miyashita H, Yamada T, et al. Risk Factors for Mortality in Patients with COVID-19 in New York City. *Journal of general internal medicine*. Published online 2020. https://link.springer.com/content/pdf/10.1007/s11606-020-05983-z.pdf

30. Okoh, Sossou C, Dangayach NS, et al. Coronavirus disease 19 in minority populations of Newark, New Jersey. *International Journal for Equity in Health*. 2020;19(1):1-8.

31. Violi F, Cangemi R, Romiti GF, et al. Is Albumin Predictor of Mortality in COVID-19? *Antioxidants & redox signaling*. Published online 2020. https://www.liebertpub.com/doi/pdf/10.1089/ars.2020.8142

32. Deng Y, Liu W, Liu K, et al. Clinical characteristics of fatal and recovered cases of coronavirus disease 2019 (COVID-19) in Wuhan, China: a retrospective study. *Chinese medical journal*. Published online 2020. https://doi.org/10.1097/cm9.0000000000000824

33. Feng Y, Ling Y, Bai T, et al. COVID-19 with Different Severities: A Multicenter Study of Clinical Features. *Am J Respir Crit Care Med*. 2020;201(11):1380-1388. doi:10.1164/rccm.202002-0445OC

34. Yang A-P, Li H-M, Tao W-Q, et al. Infection with SARS-CoV-2 causes abnormal laboratory results of multiple organs in patients. *Aging*. 2020;12. doi:10.18632/aging.103255

35. Zengin, Avcı S, Yılmaz S. Clinical and basic cardiovascular features of patients with COVID-19 admitted to a tertiary care center in Turkey. *Journal of Surgery & Medicine (JOSAM)*. 2020;4(5):367-370.

36. Wang C-Z, Hu S-L, Wang L, Li M, Li H-T. Early risk factors of the exacerbation of Coronavirus disease 2019 pneumonia. *Journal of medical virology*. Published online 2020. https://go.openathens.net/redirector/nhs?url=https%3A%2F%2Fonlinelibrary.wiley.com%2Fdoi%2Fabs%2F10.1002%2Fjmv.26071

37. Rath D, Petersen-Uribe Á, Avdiu A, et al. Impaired cardiac function is associated with mortality in patients with acute COVID-19 infection. *Clinical research in cardiology : official journal of the German Cardiac Society*. Published online 2020. https://link.springer.com/content/pdf/10.1007/s00392-020-01683-0.pdf

38. Garibaldi BT, Fiksel J, Muschelli J, et al. Patient trajectories and risk factors for severe outcomes among persons hospitalized for COVID-19 in the Maryland/DC region. *medRxiv*. Published online May 26, 2020:2020.05.24.20111864. doi:10.1101/2020.05.24.20111864

39. Yang Q, Xie L, Zhang W, et al. Analysis of the clinical characteristics, drug treatments and prognoses of 136 patients with coronavirus disease 2019. *Journal of clinical pharmacy and therapeutics*. Published online 2020. https://go.openathens.net/redirector/nhs?url=https%3A%2F%2Fonlinelibrary.wiley.com%2Fdoi%2Ffull%2F10.1111%2Fjcpt.13170

40. Petrilli CM, Jones SA, Yang J, et al. Factors associated with hospital admission and critical illness among 5279 people with coronavirus disease 2019 in New York City: prospective cohort study. *BMJ (Clinical research ed)*. 2020;369:m1966-m1966.

41. Chung SM, Lee YY, Ha E, et al. The Risk of Diabetes on Clinical Outcomes in Patients with Coronavirus Disease 2019: A Retrospective Cohort Study. *Diabetes & metabolism journal*. 2020;44(3):405-413.

42. Zhang Y, Cui Y, Shen M, et al. Association of Diabetes Mellitus with Disease Severity and Prognosis in COVID-19: A Retrospective Cohort Study. *Diabetes research and clinical practice*. Published online 2020:108227-108227.

43. M. S, U. H, P. H, et al. Coronavirus disease (COVID-19): observations and lessons from primary medical care at a German community hospital. *Journal of Community Hospital Internal Medicine Perspectives*. 2020;10(2):81-87.

44. Wang H, Lu Y, Lv Q, et al. *Progression, Recovery and Fatality in Patients with SARS-CoV-2 Related Pneumonia in Wuhan, China: A Single-Centered, Retrospective, Observational Study*. Infectious Diseases (except HIV/AIDS); 2020. doi:10.1101/2020.05.12.20099739

45. Hirsch JS, Ng JH, Ross DW, et al. Acute kidney injury in patients hospitalized with COVID-19. *Kidney International*. 2020;98(1):209-218. doi:10.1016/j.kint.2020.05.006

46. Kuno T, Takahashi M, Obata R, Maeda T. Cardiovascular comorbidities, cardiac injury and prognosis of COVID-19 in New York City. *American heart journal*. Published online 2020. https://auth.elsevier.com/ShibAuth/institutionLogin?entityID=https://idp.eng.nhs.uk/openathens&appReturnURL=https%3A%2F%2Fwww.clinicalkey.com%2Fcontent%2FplayBy%2Fdoi%2F%3Fv%3D10.1016%2Fj.ahj.2020.05.005

47. Zhang J, Ding D, Cao C, et al. *Myocardial Characteristics as the Prognosis for COVID-19 Patients*. Infectious Diseases (except HIV/AIDS); 2020. doi:10.1101/2020.05.06.20068882

48. Inciardi RM, Adamo M, Lupi L, et al. Characteristics and outcomes of patients hospitalized for COVID-19 and cardiac disease in Northern Italy. *European heart journal*. 2020;41(19):1821-1829.

49. Borobia AM, Carcas AJ, Arnalich F, et al. A cohort of patients with COVID-19 in a major teaching hospital in Europe. *medRxiv*. Published online May 6, 2020:2020.04.29.20080853. doi:10.1101/2020.04.29.20080853

50. Du R-H, Liang L-R, Yang C-Q, et al. Predictors of mortality for patients with COVID-19 pneumonia caused by SARS-CoV-2: a prospective cohort study. *The European respiratory journal*. 2020;55(5). https://erj.ersjournals.com/content/erj/early/2020/04/01/13993003.00524-2020.full.pdf

51. Hu L, Chen S, Fu Y, et al. Risk Factors Associated with Clinical Outcomes in 323 COVID-19 Hospitalized Patients in Wuhan, China. *Clinical infectious diseases : an official publication of the Infectious Diseases Society of America*. Published online 2020. https://academic.oup.com/cid/advance-article-pdf/doi/10.1093/cid/ciaa539/33156965/ciaa539.pdf

52. Wang K, Zuo P, Liu Y, et al. Clinical and Laboratory Predictors of In-hospital Mortality in Patients With Coronavirus Disease-2019: A Cohort Study in Wuhan, China. *Clin Infect Dis*. doi:10.1093/cid/ciaa538

53. Guan W, Ni Z, Hu Y, et al. Clinical Characteristics of Coronavirus Disease 2019 in China. *New England Journal of Medicine*. 2020;382(18):1708-1720. doi:10.1056/NEJMoa2002032

54. Wei J-F, Huang F-Y, Xiong T-Y, et al. Acute myocardial injury is common in patients with covid-19 and impairs their prognosis. *Heart (British Cardiac Society)*. Published online 2020.

https://go.openathens.net/redirector/nhs?url=https%3A%2F%2Fheart.bmj.com%2Flookup%2Fdoi%2F10.1136%2Fheartjnl-2020-317007

55. Wang D, Yin Y, Hu C, et al. Clinical course and outcome of 107 patients infected with the novel coronavirus, SARS-CoV-2, discharged from two hospitals in Wuhan, China. *Critical care (London, England)*. 2020;24(1):188-188.

56. Feng X, Li P, Ma L, et al. Clinical Characteristics and Short-Term Outcomes of Severe Patients with COVID-19 in Wuhan, China. *medRxiv*. Published online April 29, 2020:2020.04.24.20078063. doi:10.1101/2020.04.24.20078063

57. Zhao X-Y, Xu X-X, Yin H-S, et al. Clinical characteristics of patients with 2019 coronavirus disease in a non-Wuhan area of Hubei Province, China: a retrospective study. *BMC infectious diseases*. 2020;20(1):311-311.

58. Zhang J, Li J, Su L, et al. Clinical characteristics and risk factors of acute kidney injury in coronavirus disease 2019. *Zhonghua wei zhong bing ji jiu yi xue*. 2020;32:407-411. doi:10.3760/cma.j.cn121430-20200302-00198

59. Tomlins J, Hamilton F, Gunning S, Sheehy C, Moran E, MacGowan A. Clinical features of 95 sequential hospitalised patients with novel coronavirus 2019 disease (COVID-19), the first UK cohort. *Journal of Infection*. 2020;81(2):e59-e61. doi:10.1016/j.jinf.2020.04.020

60. Hong KS, Lee KH, Chung JH, et al. Clinical Features and Outcomes of 98 Patients Hospitalized with SARS-CoV-2 Infection in Daegu, South Korea: A Brief Descriptive Study. *Yonsei medical journal*. 2020;61(5):431-437.

61. Zhu Z, Cai T, Fan L, et al. Clinical value of immune-inflammatory parameters to assess the severity of coronavirus disease 2019. *International Journal of Infectious Diseases*. 2020;95. doi:10.1016/j.ijid.2020.04.041

62. Liu J, Li S, Liu J, et al. Longitudinal characteristics of lymphocyte responses and cytokine profiles in the peripheral blood of SARS-CoV-2 infected patients. *EBioMedicine*. 2020;55:102763. doi:10.1016/j.ebiom.2020.102763

63. X C, B Z, Y Q, et al. Detectable serum SARS-CoV-2 viral load (RNAaemia) is closely associated with drastically elevated interleukin 6 (IL-6) level in critically ill COVID-19 patients. Published online March 3, 2020. doi:10.1101/2020.02.29.20029520

64. Wang L, He WB, Yu XM, Liu HF, Zhou WJ, Jiang H. [Prognostic value of myocardial injury in patients with COVID-19]. *[Zhonghua yan ke za zhi] Chinese journal of ophthalmology*. 2020;56(0):E009-E009.

65. Li X, Xu S, Yu M, et al. Risk factors for severity and mortality in adult COVID-19 inpatients in Wuhan. *The Journal of allergy and clinical immunology*. Published online 2020. https://auth.elsevier.com/ShibAuth/institutionLogin?entityID=https://idp.eng.nhs.uk/openathens&appReturnURL=https%3A%2F%2Fwww.clinicalkey.com%2Fcontent%2FplayBy%2Fdoi%2F%3Fv%3D10.1016%2Fj.jaci.2020.04.006

66. He R, Lu Z, Zhang L, et al. The clinical course and its correlated immune status in COVID-19 pneumonia. *Journal of Clinical Virology*. 2020;127:104361. doi:10.1016/j.jcv.2020.104361

67. Fang X, Mei Q, Yang T, et al. Low-dose corticosteroid therapy does not delay viral clearance in patients with COVID-1. *Journal of Infection*. 2020;81. doi:10.1016/j.jinf.2020.03.039

68. Mao L, Jin H, Wang M, et al. Neurologic Manifestations of Hospitalized Patients With Coronavirus Disease 2019 in Wuhan, China. *JAMA Neurol*. 2020;77(6):683. doi:10.1001/jamaneurol.2020.1127

69. Gu T, Chu Q, Yu Z, et al. History of coronary heart disease increases the mortality rate of COVID-19 patients: a nested case-control study. *medRxiv*. Published online April 3, 2020:2020.03.23.20041848. doi:10.1101/2020.03.23.20041848

70. Dreher M, Kersten A, Bickenbach J, et al. The characteristics of 50 hospitalized COVID-19 patients with and without ARDS. *Deutsches Aerzteblatt Online*. 2020;117. doi:10.3238/arztebl.2020.0271

71. Wang L, Li X, Chen H, et al. Coronavirus Disease 19 Infection Does Not Result in Acute Kidney Injury: An Analysis of 116 Hospitalized Patients from Wuhan, China. *AJN*. 2020;51(5):343-348. doi:10.1159/000507471

72. Han H, Xie L, Liu R, et al. Analysis of heart injury laboratory parameters in 273 COVID-19 patients in one hospital in Wuhan, China. *Journal of medical virology*. Published online 2020. https://go.openathens.net/redirector/nhs?url=https%3A%2F%2Fonlinelibrary.wiley.com%2Fdoi%2Ffull%2F10.1002%2Fjmv.25809

73. Fan L, Liu C, Li N, et al. *Medical Treatment of 55 Patients with COVID-19 from Seven Cities in Northeast China Who Fully Recovered: A Single-Center, Retrospective, Observational Study*. Respiratory Medicine; 2020. doi:10.1101/2020.03.28.20045955

74. Li Y, Peng S, Li L, et al. Clinical and Transmission Characteristics of Covid-19 — A Retrospective Study of 25 Cases from a Single Thoracic Surgery Department. *CURR MED SCI*. 2020;40(2):295-300. doi:10.1007/s11596-020-2176-2

75. Chen G, Wu D, Guo W, et al. Clinical and immunologic features in severe and moderate forms of Coronavirus Disease 2019. *medRxiv*. Published online February 19, 2020:2020.02.16.20023903. doi:10.1101/2020.02.16.20023903

76. Guo T, Fan Y, Chen M, et al. Cardiovascular Implications of Fatal Outcomes of Patients With Coronavirus Disease 2019 (COVID-19). *JAMA cardiology*. Published online 2020. https://jamanetwork.com/journals/jamacardiology/articlepdf/2763845/jamacardiology_guo_2020_oi_200026.pdf

77. Li K, Chen D, Chen S, et al. *Radiographic Findings and Other Predictors in Adults with Covid-19*. Respiratory Medicine; 2020. doi:10.1101/2020.03.23.20041673

78. Zhou Y, Yang Z, Guo Y, et al. A New Predictor of Disease Severity in Patients with COVID-19 in Wuhan, China. :19.

79. Han Y, Zhang H, Mu S, et al. *Lactate Dehydrogenase, a Risk Factor of Severe COVID-19 Patients*. Intensive Care and Critical Care Medicine; 2020. doi:10.1101/2020.03.24.20040162

80. Shi S, Qin M, Shen B, et al. Association of Cardiac Injury With Mortality in Hospitalized Patients With COVID-19 in Wuhan, China. *JAMA cardiology*. Published online 2020. https://jamanetwork.com/journals/jamacardiology/articlepdf/2763524/jamacardiology_shi_2020_oi_200024.pdf

81. Zhang F, Yang D, Li J, et al. *Myocardial Injury Is Associated with In-Hospital Mortality of Confirmed or Suspected COVID-19 in Wuhan, China: A Single Center Retrospective Cohort Study*. Cardiovascular Medicine; 2020. doi:10.1101/2020.03.21.20040121

82. Luo X, Xia H, Yang W, et al. *Characteristics of Patients with COVID-19 during Epidemic Ongoing Outbreak in Wuhan, China*. Epidemiology; 2020. doi:10.1101/2020.03.19.20033175

83. Yan S, Song X, Lin F, et al. *Clinical Characteristics of Coronavirus Disease 2019 in Hainan, China*. Infectious Diseases (except HIV/AIDS); 2020. doi:10.1101/2020.03.19.20038539

84. Ma K-L, Liu Z-H, Cao C, et al. *COVID-19 Myocarditis and Severity Factors：An Adult Cohort Study*. Infectious Diseases (except HIV/AIDS); 2020. doi:10.1101/2020.03.19.20034124

85. Wan S, Xiang Y, Fang W, et al. Clinical features and treatment of COVID-19 patients in northeast Chongqing. *Journal of Medical Virology*. 2020;92(7):797-806. doi:10.1002/jmv.25783

86. Zheng F, Tang W, Li H, Huang Y-X, Xie Y-L, Zhou Z-G. Clinical characteristics of 161 cases of corona virus disease 2019 (COVID-19) in Changsha. *European review for medical and pharmacological sciences*. 2020;24(6):3404-3410.

87. Shi Y, Yu X, Zhao H, Wang H, Zhao R, Sheng J. Host susceptibility to severe COVID-19 and establishment of a host risk score: findings of 487 cases outside Wuhan. *Critical Care*. 2020;24(1):108. doi:10.1186/s13054-020-2833-7

88. Chen T, Wu D, Chen H, et al. Clinical characteristics of 113 deceased patients with coronavirus disease 2019: retrospective study. *BMJ (Clinical research ed)*. 2020;368:m1091-m1091.

89. liu youbin, Li J, liu D, et al. *Clinical Features and Outcomes of 2019 Novel Coronavirus-Infected Patients with Cardiac Injury*. Infectious Diseases (except HIV/AIDS); 2020. doi:10.1101/2020.03.11.20030957

90. Fu L, Fei J, Xiang H-X, et al. *Influence Factors of Death Risk among COVID-19 Patients in Wuhan, China: A Hospital-Based Case-Cohort Study*. Infectious Diseases (except HIV/AIDS); 2020. doi:10.1101/2020.03.13.20035329

91. Cao J, Tu W-J, Cheng W, et al. Clinical Features and Short-term Outcomes of 102 Patients with Coronavirus Disease 2019 in Wuhan, China. *Clin Infect Dis*. 2020;71(15):748-755. doi:10.1093/cid/ciaa243

92. Wu C, Chen X, Cai Y, et al. Risk Factors Associated With Acute Respiratory Distress Syndrome and Death in Patients With Coronavirus Disease 2019 Pneumonia in Wuhan, China. *JAMA Intern Med*. 2020;180(7):1-11. doi:10.1001/jamainternmed.2020.0994

93. Liu R, Ming X, Xu O, et al. *Association of Cardiovascular Manifestations with In-Hospital Outcomes in Patients with COVID-19: A Hospital Staff Data*. Infectious Diseases (except HIV/AIDS); 2020. doi:10.1101/2020.02.29.20029348

94. Zhou F, Yu T, Du R, et al. Clinical course and risk factors for mortality of adult inpatients with COVID-19 in Wuhan, China: a retrospective cohort study. *The Lancet*. 2020;395(10229):1054-1062. doi:10.1016/S0140-6736(20)30566-3

95. Xu H, Hou K, Xu H, et al. *Acute Myocardial Injury of Patients with Coronavirus Disease 2019*. Public and Global Health; 2020. doi:10.1101/2020.03.05.20031591

96. Zhang G, Hu C, Luo L, et al. *Clinical Features and Outcomes of 221 Patients with COVID-19 in Wuhan, China*. Respiratory Medicine; 2020. doi:10.1101/2020.03.02.20030452

97. Cao M, Zhang D, Wang Y, et al. *Clinical Features of Patients Infected with the 2019 Novel Coronavirus (COVID-19) in Shanghai, China*. Respiratory Medicine; 2020. doi:10.1101/2020.03.04.20030395

98. Chen X, Zheng F, Qing Y, et al. *Epidemiological and Clinical Features of 291 Cases with Coronavirus Disease 2019 in Areas Adjacent to Hubei, China: A Double-Center Observational Study*. Respiratory Medicine; 2020. doi:10.1101/2020.03.03.20030353

99. Bai T, Tu S, Wei Y, et al. *Clinical and Laboratory Factors Predicting the Prognosis of Patients with COVID-19: An Analysis of 127 Patients in Wuhan, China*. Social Science Research Network; 2020. doi:10.2139/ssrn.3546118

100. Yang H, Yang LC, Zhang RT, Ling YP, Ge QG. [Risks factors for death among COVID-19 patients combined with hypertension, coronary heart disease or diabetes]. *Beijing da xue xue bao Yi xue ban = Journal of Peking University Health sciences*. 2020;52(3):420-424.

101. Qi D, Yan X, Tang X, et al. Epidemiological and clinical features of 2019-nCoV acute respiratory disease cases in Chongqing municipality, China: a retrospective, descriptive, multiple-center study. *medRxiv*. Published online March 3, 2020:2020.03.01.20029397. doi:10.1101/2020.03.01.20029397

102. Wang Y, Zhou Y, Yang Z, Xia D, Geng S. *Clinical Characteristics of Patients with Severe Pneumonia Caused by the 2019 Novel Coronavirus in Wuhan, China*. Infectious Diseases (except HIV/AIDS); 2020. doi:10.1101/2020.03.02.20029306

103. Peng YD, Meng K, Guan HQ, et al. [Clinical characteristics and outcomes of 112 cardiovascular disease patients infected by 2019-nCoV]. *Zhonghua xin xue guan bing za zhi*. 2020;48(0):E004-E004.

104. Wu C, Hu X, Song J, et al. *Heart Injury Signs Are Associated with Higher and Earlier Mortality in Coronavirus Disease 2019 (COVID-19)*. Respiratory Medicine; 2020. doi:10.1101/2020.02.26.20028589

105. Zhang J, Dong X, Cao Y, et al. Clinical characteristics of 140 patients infected with SARS-CoV-2 in Wuhan, China. *Allergy*. 2020;75(7):1730-1741. doi:10.1111/all.14238

106. Liu Y, Sun W, Li J, et al. Clinical features and progression of acute respiratory distress syndrome in coronavirus disease 2019. *medRxiv*. Published online February 27, 2020:2020.02.17.20024166. doi:10.1101/2020.02.17.20024166

107. lei liu, Jian-ya G. *Clinical Characteristics of 51 Patients Discharged from Hospital with COVID-19 in Chongqing， China*. Infectious Diseases (except HIV/AIDS); 2020. doi:10.1101/2020.02.20.20025536

108. Xu M, Li M, Zhan W, et al. Clinical analysis of 23 cases of 2019 novel coronavirus infection in Xinyang City, Henan Province. *Chinese Critical Care Medicine*. Published online 2020:E010-E010.

109. Wang D, Hu B, Hu C, et al. Clinical Characteristics of 138 Hospitalized Patients With 2019 Novel Coronavirus-Infected Pneumonia in Wuhan, China. *JAMA*. Published online 2020. http://bartshealth-nhs.libsurveys.com/Barts-Health-NHS-Trust-Knowledge-and-Library-Services-Article-Request-Form

110. Huang C, Wang Y, Li X, et al. Clinical features of patients infected with 2019 novel coronavirus in Wuhan, China. *Lancet (London, England)*. 2020;395(10223):497-506.

111. Li M, Dong Y, Wang H, et al. Cardiovascular disease potentially contributes to the progression and poor prognosis of COVID-19. *Nutrition, Metabolism and Cardiovascular Diseases*. 2020;30(7):1061-1067. doi:10.1016/j.numecd.2020.04.013

112. Zhou B, She J, Wang Y, Ma X. The clinical characteristics of myocardial injury in severe and very severe patients with 2019 novel coronavirus disease. *J Infect*. 2020;81(1):147-178. doi:10.1016/j.jinf.2020.03.021

113. Zhang G, Zhang J, Wang B, Zhu X, Wang Q, Qiu S. Analysis of clinical characteristics and laboratory findings of 95 cases of 2019 novel coronavirus pneumonia in Wuhan, China: a retrospective analysis. *Respiratory Research*. 2020;21(1):74. doi:10.1186/s12931-020-01338-8

114. Hamidi Farahani R, Gholami M, Hazrati E, et al. Clinical Features of ICU Admitted and Intubated Novel Corona Virus-infected Patients in Iran. Archives of Clinical Infectious Diseases. doi:10.5812/archcid.103295

115. Klok FA, Kruip MJHA, Meer NJM van der, et al. Confirmation of the high cumulative incidence of thrombotic complications in critically ill ICU patients with COVID-19: An updated analysis. *Thrombosis Research*. 2020;191:148-150. doi:10.1016/j.thromres.2020.04.041

116. Li Y, Hu Y, Yu J, Ma T. Retrospective analysis of laboratory testing in 54 patients with severe- or critical-type 2019 novel coronavirus pneumonia. *Laboratory Investigation*. 2020;100(6):794-800. doi:10.1038/s41374-020-0431-6

117. Yang R, Gui X, Zhang Y, Xiong Y. The role of essential organ-based comorbidities in the prognosis of COVID-19 infection patients. *Expert Rev Respir Med*. 2020;14(8):835-838. doi:10.1080/17476348.2020.1761791

118. Liu M, He P, Liu HG, et al. [Clinical characteristics of 30 medical workers infected with new coronavirus pneumonia]. *Zhonghua Jie He He Hu Xi Za Zhi*. 2020;43(0):E016. doi:10.3760/cma.j.issn.1001-0939.2020.0016

119. MedetaliBeyoğlu A, Şenkal N, Çapar G, Köse M, Tükek T. Characteristics of the initial patients hospitalized for COVID-19: a single-center report. *Turk J Med Sci*.:4.

120. Ling Y, Yixiao L, Zhiping Q, et al. Clinical analysis of risk factors for severe patients with novel coronavirus pneumonia. *Chinese Journal of Infectious Diseases*. 2020;38(00):E023-E023. doi:10.3760/cma.j.cn311365-20200211-00055

121. Zhao R, Yunguang L, Yanrong L, et al. Clinical characteristics of 28 patients with novel coronavirus pneumonia. *Chinese Journal of Infectious Diseases*. 2020;38(00):E006-E006. doi:10.3760/cma.j.issn.1000-6680.2020.0006

122. Ma Y, Diao B, Lv X, et al. Epidemiological, Clinical, and Immunological Features of a Cluster of COVID-19–Contracted Hemodialysis Patients. *Kidney International Reports*. 2020;5(8):1333-1341. doi:10.1016/j.ekir.2020.06.003

123. Yaghi Shadi, Ishida Koto, Torres Jose, et al. SARS-CoV-2 and Stroke in a New York Healthcare System. *Stroke*. 2020;51(7):2002-2011. doi:10.1161/STROKEAHA.120.030335

124. Fu S, Fu X, Song Y, et al. *Virologic and Clinical Characteristics for Prognosis of Severe COVID-19: A Retrospective Observational Study in Wuhan, China*. Infectious Diseases (except HIV/AIDS); 2020. doi:10.1101/2020.04.03.20051763

125. Colon CM, Barrios JG, Chiles JW, et al. Atrial Arrhythmias in COVID-19 Patients. *JACC: Clinical Electrophysiology*. 2020;6(9):1189-1190. doi:10.1016/j.jacep.2020.05.015

126. Liu K, Fang Y-Y, Deng Y, et al. Clinical characteristics of novel coronavirus cases in tertiary hospitals in Hubei Province. *Chinese Medical Journal*. 2020;133(9):1025-1031. doi:10.1097/CM9.0000000000000744

127. Zhu Y, Du Z, Zhu Y, Li W, Miao H, Li Z. Evaluation of organ function in patients with severe COVID-19 infections. *Medicina Clínica*. 2020;155(5):191-196. doi:10.1016/j.medcli.2020.05.012

128. Cantador E, Núñez A, Sobrino P, et al. Incidence and consequences of systemic arterial thrombotic events in COVID-19 patients. *J Thromb Thrombolysis*. 2020;50(3):543-547. doi:10.1007/s11239-020-02176-7

129. Szekely Yishay, Lichter Yael, Taieb Philippe, et al. Spectrum of Cardiac Manifestations in COVID-19. *Circulation*. 2020;142(4):342-353. doi:10.1161/CIRCULATIONAHA.120.047971

130. Sud K, Vogel B, Bohra C, et al. Echocardiographic Findings in Patients with COVID-19 with Significant Myocardial Injury. *Journal of the American Society of Echocardiography*. 2020;33(8):1054-1055. doi:10.1016/j.echo.2020.05.030

131. Jain SS, Liu Q, Raikhelkar J, et al. Indications for and Findings on Transthoracic Echocardiography in COVID-19. *Journal of the American Society of Echocardiography*. 2020;33(10):1278-1284. doi:10.1016/j.echo.2020.06.009

132. Li J, Zhang Y, Wang F, et al. *Sex Differences in Clinical Findings among Patients with Coronavirus Disease 2019 (COVID-19) and Severe Condition*. Respiratory Medicine; 2020. doi:10.1101/2020.02.27.20027524

133. Chen C, Chen C, Yan JT, Zhou N, Zhao JP, Wang DW. [Analysis of myocardial injury in patients with COVID-19 and association between concomitant cardiovascular diseases and severity of COVID-19]. *Zhonghua Xin Xue Guan Bing Za Zhi*. 2020;48(7):567-571. doi:10.3760/cma.j.cn112148-20200225-00123

134. Sun C, Zhang XB, Dai Y, Xu XZ, Zhao J. [Clinical analysis of 150 cases of 2019 novel coronavirus infection in Nanyang City, Henan Province]. *Zhonghua Jie He He Hu Xi Za Zhi*. 2020;43(6):503-508. doi:10.3760/cma.j.cn112147-20200224-00168

135. Sabatino J, Ferrero P, Chessa M, et al. COVID-19 and Congenital Heart Disease: Results from a Nationwide Survey. *Journal of Clinical Medicine*. 2020;9(6):1774. doi:10.3390/jcm9061774

136. López-Otero D, López-Pais J, Cacho-Antonio CE, et al. Impact of angiotensin-converting enzyme inhibitors and angiotensin receptor blockers on COVID-19 in a western population. CARDIOVID registry. *Revista Española de Cardiología (English Edition)*. Published online June 5, 2020. doi:10.1016/j.rec.2020.05.018

137. Escalera-Antezana JP, Lizon-Ferrufino NF, Maldonado-Alanoca A, et al. Risk factors for mortality in patients with Coronavirus Disease 2019 (COVID-19) in Bolivia: An analysis of the first 107 confirmed cases. *Infez Med*. 2020;28(2):238-242.

138. Gao Q, Hu Y, Dai Z, Xiao F, Wang J, Wu J. The epidemiological characteristics of 2019 novel coronavirus diseases (COVID-19) in Jingmen, Hubei, China. *Medicine*. 2020;99(23):e20605. doi:10.1097/MD.0000000000020605

139. Kim DW, Byeon KH, Kim J, Cho KD, Lee N. The Correlation of Comorbidities on the Mortality in Patients with COVID-19: an Observational Study Based on the Korean National Health Insurance Big Data. *J Korean Med Sci*. 2020;35(26):e243. doi:10.3346/jkms.2020.35.e243

140. Luo Y, Xue Y, Mao L, et al. Prealbumin as a Predictor of Prognosis in Patients With Coronavirus Disease 2019. *Front Med*. 2020;7:374. doi:10.3389/fmed.2020.00374

141. Garcia PDW, Fumeaux T, Guerci P, et al. Prognostic factors associated with mortality risk and disease progression in 639 critically ill patients with COVID-19 in Europe: Initial report of the international RISC-19-ICU prospective observational cohort. *EClinicalMedicine*. 2020;25. doi:10.1016/j.eclinm.2020.100449

142. Ruan Q, Yang K, Wang W, Jiang L, Song J. Clinical predictors of mortality due to COVID-19 based on an analysis of data of 150 patients from Wuhan, China. *Intensive Care Med*. 2020;46(5):846-848. doi:10.1007/s00134-020-05991-x

143. Liu J, Liu Y, Xiang P, et al. Neutrophil-to-Lymphocyte Ratio Predicts Severe Illness Patients with 2019 Novel Coronavirus in the Early Stage. *medRxiv*. Published online February 12, 2020:2020.02.10.20021584. doi:10.1101/2020.02.10.20021584

144. Hui H, Zhang Y, Yang X, et al. *Clinical and Radiographic Features of Cardiac Injury in Patients with 2019 Novel Coronavirus Pneumonia*. Cardiovascular Medicine; 2020. doi:10.1101/2020.02.24.20027052

145. Palmieri L, Vanacore N, Donfrancesco C, et al. Clinical Characteristics of Hospitalized Individuals Dying With COVID-19 by Age Group in Italy. *J Gerontol A Biol Sci Med Sci*. 2020;75(9):1796-1800. doi:10.1093/gerona/glaa146

146. Bhatraju PK, Ghassemieh BJ, Nichols M, et al. Covid-19 in Critically Ill Patients in the Seattle Region — Case Series. *New England Journal of Medicine*. Published online March 30, 2020. Accessed October 17, 2020. https://www.nejm.org/doi/10.1056/NEJMoa2004500

147. Fan H, Zhang L, Huang B, et al. Cardiac injuries in patients with coronavirus disease 2019: Not to be ignored. *International Journal of Infectious Diseases*. 2020;96:294-297. doi:10.1016/j.ijid.2020.05.024

148. Zheng Y, Sun L, Xu M, et al. Clinical characteristics of 34 COVID-19 patients admitted to intensive care unit in Hangzhou, China. *J Zhejiang Univ Sci B*. 2020;21(5):378-387. doi:10.1631/jzus.B2000174

149. Yang X, Yu Y, Xu J, et al. Clinical course and outcomes of critically ill patients with SARS-CoV-2 pneumonia in Wuhan, China: a single-centered, retrospective, observational study. *The Lancet Respiratory Medicine*. 2020;8(5):475-481. doi:10.1016/S2213-2600(20)30079-5

150. Yu Y, Xu D, Fu S, et al. Patients with COVID-19 in 19 ICUs in Wuhan, China: a cross-sectional study. *Critical Care*. 2020;24(1):219. doi:10.1186/s13054-020-02939-x

151. Ge H, Zhu M, Du J, et al. Cardiac Structural and Functional Characteristics in Patients with Coronavirus Disease 2019: A Serial Echocardiographic Study. *medRxiv*. Published online May 18, 2020:2020.05.12.20095885. doi:10.1101/2020.05.12.20095885

152. Xu Y, Xu Z, Liu X, et al. Clinical findings in critical ill patients infected with SARS-Cov-2 in Guangdong Province, China: a multi-center, retrospective, observational study. *medRxiv*. Published online March 6, 2020:2020.03.03.20030668. doi:10.1101/2020.03.03.20030668

153. Zou X, Li S, Fang M, et al. Acute Physiology and Chronic Health Evaluation II Score as a Predictor of Hospital Mortality in Patients of Coronavirus Disease 2019. *Critical Care Medicine*. 2020;48(8):e657. doi:10.1097/CCM.0000000000004411

154. Xu J, Yang X, Yang L, et al. Clinical course and predictors of 60-day mortality in 239 critically ill patients with COVID-19: a multicenter retrospective study from Wuhan, China. *Critical Care*. 2020;24(1):394. doi:10.1186/s13054-020-03098-9

155. Shang Y, Liu T, Wei Y, et al. Scoring systems for predicting mortality for severe patients with COVID-19. *EClinicalMedicine*. 2020;24. doi:10.1016/j.eclinm.2020.100426

156. Ferrando C, Mellado-Artigas R, Gea A, et al. Patient characteristics, clinical course and factors associated to ICU mortality in critically ill patients infected with SARS-CoV-2 in Spain: A prospective, cohort, multicentre study. *Revista Española de Anestesiología y Reanimación (English Edition)*. Published online July 11, 2020. doi:10.1016/j.redare.2020.07.001

157. Hu B. Clinical features of critically ill patients with COVID-19 infection in China. Published online March 8, 2020. doi:10.21203/rs.3.rs-16250/v1

158. Zhou W, Liu Y, Tian D, et al. Potential benefits of precise corticosteroids therapy for severe 2019-nCoV pneumonia. *Signal Transduction and Targeted Therapy*. 2020;5(1):1-3. doi:10.1038/s41392-020-0127-9

159. Shi S, Qin M, Cai Y, et al. Characteristics and clinical significance of myocardial injury in patients with severe coronavirus disease 2019. *Eur Heart J*. 2020;41(22):2070-2079. doi:10.1093/eurheartj/ehaa408

160. He X, Jinsheng L, Jia C, et al. Impact of complicated myocardial injury on the clinical outcome of severe or critically ill COVID-19 patients. *Chinese Journal of Cardiology*. 2020;48(00):E011-E011. doi:10.3760/cma.j.cn112148-20200228-00137

161. Li J, Xu G, Yu H, Peng X, Luo Y, Cao C. Clinical Characteristics and Outcomes of 74 Patients With Severe or Critical COVID-19. *The American Journal of the Medical Sciences*. 2020;360(3):229-235. doi:10.1016/j.amjms.2020.05.040

162. Yan Y, Yang Y, Wang F, et al. Clinical characteristics and outcomes of patients with severe covid-19 with diabetes. *BMJ Open Diabetes Research and Care*. 2020;8(1):e001343. doi:10.1136/bmjdrc-2020-001343

163. Huang M, Yang Y, Shang F, et al. Clinical Characteristics and Predictors of Disease Progression in Severe Patients with COVID-19 Infection in Jiangsu Province, China: A Descriptive Study. *The American Journal of the Medical Sciences*. 2020;360(2):120-128. doi:10.1016/j.amjms.2020.05.038

164. Cummings MJ, Baldwin MR, Abrams D, et al. Epidemiology, clinical course, and outcomes of critically ill adults with COVID-19 in New York City: a prospective cohort study. *The Lancet*. 2020;395(10239):1763-1770. doi:10.1016/S0140-6736(20)31189-2

165. Chen Q, Xu L, Dai Y, et al. Cardiovascular manifestations in severe and critical patients with COVID-19. *Clinical Cardiology*. 2020;43(7):796-802. doi:10.1002/clc.23384

166. Xu Z, Wang Z, Wang S, et al. The impact of type 2 diabetes and its management on the prognosis of patients with severe COVID-19. *Journal of Diabetes*. n/a(n/a). doi:10.1111/1753-0407.13084

167. Pan F, Yang L, Li Y, et al. Factors associated with death outcome in patients with severe coronavirus disease-19 (COVID-19): a case-control study. *Int J Med Sci*. 2020;17(9):1281-1292. doi:10.7150/ijms.46614

168. Chen F, Zhong M, Liu Y, et al. The characteristics and outcomes of 681 severe cases with COVID-19 in China. *Journal of Critical Care*. 2020;60:32-37. doi:10.1016/j.jcrc.2020.07.003

169. Arentz M, Yim E, Klaff L, et al. Characteristics and Outcomes of 21 Critically Ill Patients With COVID-19 in Washington State. *JAMA*. 2020;323(16):1612-1614. doi:10.1001/jama.2020.4326

170. Wang Y, Lu X, Li Y, et al. Clinical Course and Outcomes of 344 Intensive Care Patients with COVID-19. *Am J Respir Crit Care Med*. 2020;201(11):1430-1434. doi:10.1164/rccm.202003-0736LE

171. Yang F, Shi S, Zhu J, Shi J, Dai K, Chen X. Analysis of 92 deceased patients with COVID-19. *Journal of Medical Virology*. 2020;92(11):2511-2515. doi:10.1002/jmv.25891

172. Du Y, Tu L, Zhu P, et al. Clinical Features of 85 Fatal Cases of COVID-19 from Wuhan. A Retrospective Observational Study. *Am J Respir Crit Care Med*. 2020;201(11):1372-1379. doi:10.1164/rccm.202003-0543OC

173. Shi Q, Zhao K, Yu J, et al. Clinical characteristics of 101 COVID-19 nonsurvivors in Wuhan, China: a retrospective study. *medRxiv*. Published online May 20, 2020:2020.03.04.20031039. doi:10.1101/2020.03.04.20031039

174. Zhang B, Zhou X, Qiu Y, et al. Clinical characteristics of 82 death cases with COVID-19. *medRxiv*. Published online February 27, 2020:2020.02.26.20028191. doi:10.1101/2020.02.26.20028191

175. Li X, Wang L, Yan S, et al. Clinical characteristics of 25 death cases with COVID-19: A retrospective review of medical records in a single medical center, Wuhan, China. *International Journal of Infectious Diseases*. 2020;94:128-132. doi:10.1016/j.ijid.2020.03.053

176. Huang Y, Yang R, Xu Y, Gong P. Clinical characteristics of 36 non-survivors with COVID-19 in Wuhan, China. *medRxiv*. Published online March 5, 2020:2020.02.27.20029009. doi:10.1101/2020.02.27.20029009

177. Du R-H, Liu L-M, Yin W, et al. Hospitalization and Critical Care of 109 Decedents with COVID-19 Pneumonia in Wuhan, China. *Ann Am Thorac Soc*. 2020;17(7):839-846. doi:10.1513/AnnalsATS.202003-225OC

178. Gao L, Jiang D, Wen X, et al. Prognostic value of NT-proBNP in patients with severe COVID-19. *Respiratory Research*. 2020;21(1):83. doi:10.1186/s12931-020-01352-w

179. Palaiodimos L, Kokkinidis DG, Li W, et al. Severe obesity, increasing age and male sex are independently associated with worse in-hospital outcomes, and higher in-hospital mortality, in a cohort of patients with COVID-19 in the Bronx, New York. *Metabolism*. 2020;108:154262. doi:10.1016/j.metabol.2020.154262

180. Zhao M, Wang M, Zhang J, et al. Comparison of clinical characteristics and outcomes of patients with coronavirus disease 2019 at different ages. *Aging*. 2020;12(11):10070-10086. doi:10.18632/aging.103298

181. Li P, Chen L, Liu Z, et al. Clinical features and short-term outcomes of elderly patients with COVID-19. *International Journal of Infectious Diseases*. 2020;97:245-250. doi:10.1016/j.ijid.2020.05.107

182. Wang L, He W, Yu X, et al. Coronavirus disease 2019 in elderly patients: Characteristics and prognostic factors based on 4-week follow-up. *Journal of Infection*. 2020;80(6):639-645. doi:10.1016/j.jinf.2020.03.019

183. Li T, Lu L, Zhang W, et al. Clinical characteristics of 312 hospitalized older patients with COVID-19 in Wuhan, China. *Archives of Gerontology and Geriatrics*. 2020;91:104185. doi:10.1016/j.archger.2020.104185

184. Wang F, Yang Y, Dong K, et al. Clinical characteristics of 28 patients with diabetes and covid-19 in wuhan, china. *Endocrine Practice*. 2020;26(6):668-674. doi:10.4158/EP-2020-0108

185. Li Y, Han X, Alwalid O, et al. Baseline characteristics and risk factors for short-term outcomes in 132 COVID-19 patients with diabetes in Wuhan China: A retrospective study. *Diabetes Res Clin Pract*. 2020;166:108299. doi:10.1016/j.diabres.2020.108299

186. Louhaichi S, Allouche A, Baili H, et al. Features of patients with 2019 novel coronavirus admitted in a pneumology department: The first retrospective Tunisian case series. *Tunis Med*. 2020;98(4):261-265.

187. Aggarwal S, Garcia-Telles N, Aggarwal G, Lavie C, Lippi G, Henry BM. Clinical features, laboratory characteristics, and outcomes of patients hospitalized with coronavirus disease 2019 (COVID-19): Early report from the United States. *Diagnosis*. 2020;7(2):91-96. doi:10.1515/dx-2020-0046

188. Su M, Peng J, Wu M, Deng W, Yang Y, Peng YG. Two consecutive myocardial tissue insults for inpatients with COVID-19. *Critical Care*. 2020;24(1):259. doi:10.1186/s13054-020-02981-9

189. Yang F, Shi S, Zhu J, Shi J, Dai K, Chen X. Clinical characteristics and outcomes of cancer patients with COVID-19. *Journal of Medical Virology*. 2020;92(10):2067-2073. doi:10.1002/jmv.25972

190. Xie H, Zhao J, Lian N, Lin S, Xie Q, Zhuo H. Clinical characteristics of non-ICU hospitalized patients with coronavirus disease 2019 and liver injury: A retrospective study. *Liver International*. 2020;40(6):1321-1326. doi:10.1111/liv.14449

191. Si D, Du B, Ni L, et al. Death, discharge and arrhythmias among patients with COVID-19 and cardiac injury. *CMAJ*. 2020;192(28):E791-E798. doi:10.1503/cmaj.200879

192. Pierce-Williams RAM, Burd J, Felder L, et al. Clinical course of severe and critical coronavirus disease 2019 in hospitalized pregnancies: a United States cohort study. *American Journal of Obstetrics & Gynecology MFM*. 2020;2(3). doi:10.1016/j.ajogmf.2020.100134
